# Enhanced Efficacy of Rhizosphere Microorganisms and Green Compounds: A Dual-Action Strategy Against *Bursaphelenchus xylophilus* in *Pinus massoniana*

**DOI:** 10.3390/microorganisms14061202

**Published:** 2026-05-26

**Authors:** Jiacheng Zhu, Yi Dang, Xiaoming Ren, Long Xu, Yilong Zhou, Guoying Zhou, Junang Liu

**Affiliations:** 1 College of Forestry, Central South University of Forestry and Technology, Changsha 410004, China; 2 Hunan Provincial Key Laboratory for Control of Forest Diseases and Pestsand Pests in South China, Central South University of Forestry and Technology, Changsha 410004, China; 3 Key Laboratory for Non-Wood Forest Cultivation and Conservation of Ministry of Education, Central South University of Forestry and Technology, Changsha 410004, China; 4 Yuelushan Laboratory Non-Wood Forests Variety Innovation Center, Central South University of Forestry and Technology, Changsha 410004, China

**Keywords:** pine wilt disease, microbial metabolites, defense response mechanisms, green agent formulation

## Abstract

Effective and sustainable control strategies for pine wilt disease, caused by the pine wood nematode (*Bursaphelenchus xylophilus*), are urgently needed, as reliance on conventional chemical nematicides faces increasing limitations. In this study, a new kind of integrated approach is proposed. It pairs microbial fermentation filtrates with the green chemicals arecoline and sodium silicate. The filtrates were obtained from bacterial and fungal strains that were had isolated from *Pinus massoniana* rhizosphere soil. The nematicidal efficacy of individual and combined treatments was evaluated in vitro, while their ability to induce systemic resistance in *P. massoniana* seedlings was assessed through defense enzyme assays, malondialdehyde (MDA) content measurement, and defense-related gene expression analysis. Results identified several highly effective combinations, particularly arecoline plus CSZ33 and sodium silicate plus CSUFT-F23, which achieved over 72% control efficacy. These formulations not only showed direct toxicity but also significantly enhanced the plant’s antioxidant capacity and upregulated key defense genes. Furthermore, untargeted metabolomics linked these effects to specific bioactive metabolites in the fermentation filtrates, such as D-glutamic acid. This work demonstrates that hybrid bio-chemical formulations can successfully merge immediate pathogen suppression with long-term host resistance priming, offering a promising, sustainable strategy for the integrated management of pine wilt disease.

## 1. Introduction

Pine wilt disease (PWD), caused by the pine wood nematode (*B. xylophilus*), is a devastating threat to global pine forests [1,2,3,4,5]. Often termed a “forest fire without smoke,” it can kill a mature pine tree within months, leading to massive ecological and economic losses [6,7]. Since its introduction to China in 1982, PWD has caused the death of billions of trees, resulting in incalculable damage to forest ecosystems and staggering economic costs [8,9,10,11]. Containing its spread remains a critical challenge for forestry protection. Current management strategies have significant limitations. Strict quarantine and the removal of infected trees are essential but often reactive and resource-intensive [12,13,14,15]. Chemical nematicides, while effective, raise serious concerns about environmental pollution, pesticide residues, and the development of resistance [16,17]. In contrast, biological control using microbial agents like bacteria or fungi offers an environmentally friendly alternative [18]. However, its real-world application is often hampered by inconsistent field efficacy, slow action, and susceptibility to environmental conditions [19,20]. A promising yet underexplored avenue is the development of combined formulations that integrate biological and chemical agents to achieve enhanced efficacy, potentially enhancing efficacy while reducing the drawbacks of each component alone [21,22,23].

For plant-parasitic nematode control, hybrid and integrated formulations offer clear advantages over single-method strategies by addressing poor stability, environmental sensitivity, and resistance selection [24,25,26]. These formulations combine biocontrol agents, low-dose chemical nematicides, encapsulated plant metabolites, and diverse rhizosphere microbes to achieve metabolic complementarity and multi-target activity [27,28,29]. Integrating complementary modes of action with optimized component compatibility leads to broader and more consistent nematode suppression under both greenhouse and field conditions [30,31]. Despite the recognized potential of combined strategies, research specifically targeting PWD with hybrid bio-chemical formulations is scarce [32,33]. Most studies focus on screening single microbial strains or chemical agents [34,35]. There is a distinct lack of systematic investigation into how fermentation filtrates from promising biocontrol microbes can be effectively paired with green chemical nematicides [36,37]. Furthermore, the underlying mechanisms of such combinations particularly their role in inducing systemic resistance in pine trees are poorly understood [38,39,40]. This knowledge gap hinders the development of reliable, potent, and sustainable control agents. To address these shortcomings, this study aimed to develop and evaluate novel green combined formulations for PWD management. Building on previous research [36,41], this study focuses on the integration of selected microbial fermentation filtrates (bacterial and fungal strains isolated from the rhizosphere soil of *Pinus massoniana*) with effective green chemical substances (arecoline and sodium silicate). The objectives were threefold: (1) to screen and identify high-performance individual components, (2) to formulate combined agents and assess their direct nematicidal efficacy against *B. xylophilus*, and (3) to investigate their ability to induce defense responses in *P. massoniana* seedlings. The present work employed a multi-method approach, including in vitro nematicidal assays, measurements of defense-related enzyme activities and malondialdehyde content, analysis of defense gene expression, and untargeted metabolomics to profile the active components in the microbial filtrates.

Results demonstrate that specific combinations, particularly arecoline plus CSZ33 (*Penicillium sclerotiorum*) and sodium silicate plus CSUFT-F23 (*Tolypocladium* sp.), exhibit superior performance. They achieved high direct mortality against nematodes (>72% control efficacy) while simultaneously priming the pine seedlings’ immune system, as evidenced by enhanced antioxidant enzyme activities and upregulated expression of key defense genes. Metabolomics further linked these effects to specific bioactive metabolites.

## 2. Materials and Methods

### 2.1. Microbial Strains

The strains of fungal and bacteria active against the pine wood nematode are maintained in the laboratory and were isolated from soil around the roots of *P. massoniana* trees that had not been treated with any chemicals [36,41].The strain of *Botrytis cinerea* used to feed *B. xylophilus* in this experiment was obtained from Zhejiang University Agriculture and Forestry. The strain is held in the laboratory repository.

### 2.2. Pine Wood Nematode

The pathogen *B. xylophilus*, responsible for pine wilt disease (PWN), was isolated from diseased *P. massoniana* trees using a Behrman funnel [40], long-term cultivation.

### 2.3. Medium

Bacterial medium: Luria–Bertani (LB); fungal medium: potato dextrose agar (PDA) [36,41]; formulation details are provided in Supplementary Material File S2.

### 2.4. Fermentation Filtrates of Tested Strains

For bacterial strains: on a clean bench, 100 μL of stock culture was transferred into a sterile 1.5 mL tube containing 1 mL of liquid LB medium [36,41]. The culture was incubated in a shaking incubator at 37 °C in the dark for one day. Subsequently, the bacterial suspension was diluted with sterile water and spread onto solid LB plates, followed by incubation at 37 °C. After confirming no microbial growth on the control plates, the nematicidal assays were conducted. Single colonies were picked and transferred. Fresh stock cultures were prepared from them. Subsequently, 7.5 mL of this culture were transferred into a flask containing 150 mL of liquid LB and incubated at 37 °C with shaking at 180 rpm for three days. Following incubation, the culture was centrifuged at 12,000 rpm for 5 min at 4 °C, and the supernatant was filtered through a 0.22 μm filter. The resulting filtrate was used as the bacterial fermentation filtrate.

For fungal isolates: solid PDA [36,41] was poured into 90 mm Petri dishes. After solidification, fungal cultures were transferred from storage tubes onto the plates and incubated in the dark at 28 °C. After seven days, the cultures were subcultured again. Once the plates were fully colonized, a sterile cork borer was used to cut out 5 mm mycelial plugs, which were then transferred into flasks containing 150 mL of liquid PDA medium. These were incubated in a shaking incubator at 28 °C and 180 rpm for five days. After incubation, mycelia were removed by filtering through sterile gauze, and the liquid was collected in centrifuge tubes. The supernatant was separated by centrifugation for 5 min and then filtered through a 0.45 μm filter to obtain the fungal fermentation filtrate.

### 2.5. Green Low-Toxicity Agents for Testing

Eight concentrations (0.25, 0.5, 1, 1.5, 2, 2.5, 3, and 4 g/L) of each test agent were prepared in deionized water, and their nematicidal activity against *B. xylophilus* was evaluated. The optimal concentration was determined based on the frequency of improvement in nematicidal activity with increasing concentration [42].

### 2.6. Screening for High-Efficacy Nematicidal Strains and Green Low-Toxicity Agents

Nematode suspensions (500 μL) were mixed 1:1 with either the fermentation filtrate of the test strain or one of the eight agent concentrations. The mixtures were incubated at 25 °C in the dark. Nematode viability was assessed under a stereomicroscope at 24, 48, and 72 h. Nematodes showing typical serpentine (S shaped) or spiral movement were counted as alive. Those that remained motionless with a J- or C-shaped body, or appeared rigid with a loss of body wall refractivity, were counted as dead [43,44,45].

Corrected mortality was calculated as

Corrected mortality (%) = [(treatment mortality − control mortality)/(1 − control mortality)] × 100

### 2.7. Green Combined Formulations Against B. xylophilus

The selected bacterial strains were inoculated into 150 mL of liquid LB medium and cultured on a shaker at 37 °C and 180 rpm for 72 h. The cultures were then centrifuged, and the supernatants were filtered through 0.22 μm membrane filters to obtain the bacterial fermentation filtrates. For the fungal strains, each was inoculated into 150 mL of liquid PDA medium and incubated on a shaker at 28 °C and 180 rpm for five days. After centrifugation, the supernatants were filtered through 0.45 μm membrane filters to obtain the fungal fermentation filtrates. Arecoline, sodium silicate, and ginkgo extract were used. These were selected based on the screening results. Each compound was dissolved in deionized water. Solutions were prepared at 1.5 g/L. The selected bacterial or fungal fermentation filtrates were then combined with the green nematicidal agents in a 1:1 ratio, forming either bacterium–agent or fungus–agent mixtures. Nematicidal activity was assessed following the procedure described in Section 2.5.

### 2.8. Green Agent Pot Trial on P. massoniana Seedlings Inoculated with B. xylophilus

Two-year-old *P. massoniana* seedlings were acclimatized for two weeks. After acclimation, 42 healthy, uniformly growing seedlings were selected, 6 of which served as controls. Treatments including fermentation filtrates, nematicidal agents, and their combinations were applied to the soil using a root-drench method. For single-filtrate treatments, 50 mL of filtrate was applied. For single-agent treatments, 50 mL of the aqueous solution was used. For combined treatments, 50 mL of a 1:1 mixture was applied. Following treatment, *B. xylophilus* was inoculated onto the seedlings using the bark grafting method [46,47]. The inoculation site was wrapped with absorbent cotton, sealed with plastic film and a plastic dropper tip to prevent leakage. Each treatment group (single filtrate, single agent, combined formulation, and the nematode-only control (CK1)) received 2 mL of nematode suspension containing approximately 3000 nematodes/mL. The blank control (CK) received 2 mL of sterile water. Three days later, all treatment groups received a second root-drench application [48,49]. Details of the treatments are provided in Supplementary Material File S2.

### 2.9. Assay of Defense Enzyme and MDA Activities in P. massoniana Seedlings

All kits, including the ascorbate peroxidase (APX) assay kit (G0203F), glutathione reductase (GR) assay kit (G0209F), polyphenol oxidase (PPO) assay kit (G0113F), and malondialdehyde (MDA) assay kit (G0109F) were obtained from Suzhou Grace Biotechnology, Suzhou, China [50,51,52]. The methods for measuring defense enzyme activities and malondialdehyde (MDA) content are detailed in Supplementary Material File S2.

### 2.10. Control Efficacy of Combined Green Formulations Against Pine Wilt Disease in P. massoniana Seedlings

After 28 days of treatment, the disease index of *P. massoniana* seedlings was monitored and evaluated according to a disease rating scale. Control efficacy was then calculated for each treatment group [46].

Disease severity index (DSI) = [Σ(number of diseased branches × disease rating value)/(total number of branches × maximum disease grade)] × 100

The disease rating criteria for pine wilt disease are detailed in Supplementary Material File S2.

### 2.11. Detection of Defense Related Gene Expression in P. massoniana by Retrotranscribed Quantitative PCR

For analysis of defense-related gene expression, two treatment combinations, arecoline plus CSZ33 and sodium silicate plus CSUFT-F23, were selected. Twenty-four healthy, uniformly growing *P. massoniana* seedlings were used, six of which served as controls. The treatments were as follows: CK and CK1 groups received a root drench of 50 mL sterile water. The two combination groups each received 50 mL of a 1:1 mixture of the corresponding fermentation filtrate and green agent solution [53,54].

All groups except CK were then inoculated with *B. xylophilus* using the bark grafting method described in Section 2.8. Needle samples were collected on days 1, 3, 5, 7, and 14 after inoculation for total RNA extraction. Three biological replicates were sampled per treatment at each time point. Total RNA was extracted using the Polysaccharide Polyphenol Plant Total RNA Extraction Kit (DP441, Tiangen Biotech, Beijing, China), following the manufacturer’s instructions with minor adjustments tailored to the sample type and experimental conditions. First-strand cDNA was synthesized from the extracted RNA using a reverse transcription premix kit (AG11728, Accurate Biotechnology, Hunan, Changsha, China). Retrotranscribed quantitative PCR was performed on a QuantStudio 3 system using 2× SYBR Green qPCR Mix (AH0104, Scientech, Jinan, China). Cycling conditions followed the kit protocol. Gene expression levels were calculated using the 2^−ΔΔCT^ method, with actin serving as the internal reference gene [54,55]. Primers used for amplification are listed in Supplementary Material File S2 and were synthesized by Shanghai Sangon Biotech, Shanghai, China.

### 2.12. LC-MS Sample Preparation

Fermentation filtrates of the four strains were prepared as described in Section 2.3. For each sample, 100 μL of the filtrate was transferred to a 1.5 mL centrifuge tube and mixed with 400 μL of extraction buffer. The mixture was vortexed for 30 s, then subjected to low-temperature ultrasonication for 30 min. After extraction, samples were left to stand at −20 °C for 30 min, followed by centrifugation at low temperature for 15 min. The supernatant was collected and evaporated to dryness under nitrogen. The residue was reconstituted in 100 μL of reconstitution solution, extracted by ultrasonication at 4 °C for 5 min, and centrifuged for 10 min. The final supernatant was transferred to a sample vial for analysis by liquid chromatography–tandem mass spectrometry (LC-MS/MS).

### 2.13. LC-MS Analysis

The samples were analyzed by LC-MS/MS by Shanghai Meiji Biotechnology, Shanghai, China.

### 2.14. Assaying Metabolites for Nematicidal Activity

The selected metabolites were tested for nematicidal activity using an immersion method. The assay procedure was the same as that described for evaluating nematicidal activity in Section 2.5.

### 2.15. Statistical Analysis

Nematicidal activity was classified according to the criteria described by Chandravadana [55], with details provided in Supplementary Material File S2. Statistical analyses were performed using Microsoft Excel for data compilation and calculations. Differences among treatments were evaluated for significance using IBM SPSS Statistics 27, with significance set at *p* < 0.05. Graphical illustrations were generated with Origin 2021.

Raw LC-MS data were preprocessed using the metabolomics software Progenesis QI, v3.0 (Waters Corporation, Milford, MA, USA), and the resulting three-dimensional data matrices were exported in CSV format. The data matrices were then uploaded to the Majorbio Cloud Platform (www.cloud.majorbio.com) provided by Shanghai Majorbio Bio-Pharm Technology, Shanghai, China, for further analysis.

The detailed methods are provided in Supplementary Material File S2.

## 3. Results

### 3.1. Screening of Active Strains and Chemical Agents for Killing Pine Wood Nematodes

From an initial screening of 12 bacterial and 12 fungal fermentation filtrates (Tables S1 and S2), the present work identified several isolates with strong nematicidal activity against *B. xylophilus*. The performance of isolate CSX134 was striking. It remained strong across all time points. By 72 h, corrected mortality reached over 90%. CSX30 came in a close second. Among the fungi, CSZ71 was the top performer, with mortality rates above 94% at 72 h, followed closely by CSZ33. Interestingly, while most strains showed time-dependent activity, the rate of increase tended to slow after 48 h, and a few strains like CSUFT-B60 and CSUFT-F48 showed only marginal improvements throughout.

This study also screened eleven environmentally friendly substances for nematicidal potential. The corresponding data are presented in Figure S1. All of them exhibited clear dose and time-dependent effects. Arecoline, sodium silicate, and ginkgo extract were the most effective. Arecoline’s activity jumped noticeably between 24 and 48 h, and at 4 g/L it reached 92.6% mortality by 72 h. Sodium silicate was consistently strong across concentrations, hitting over 60% mortality even at 24 h. Ginkgo extract, while slower to start, still reached nearly 90% at the highest concentration.

Balancing efficacy with safety and environmental concerns (Table S3), the present work settled on 1.5 g/L as the working concentration for subsequent experiments. At this level, most agents showed solid activity, sodium silicate and arecoline performed particularly well by 72 h, and even ginkgo extract, despite its slower start, reached 70% mortality. Based on these results, sodium silicate, arecoline, and ginkgo extract were selected as the preferred bioactive agents for the next experimental phase.

### 3.2. Green Compound Formulation for Killing Pine Wood Nematodes

The present work then tested pairings of bacterial or fungal fermentation filtrates with the chosen green substances. For most combinations, the effect was clearly time-dependent. Mortality generally peaked at 72 h. For bacterial-based mixes (Table S4), arecoline plus CSX134 was the standout. It started strong at 24 h (69.89%) and climbed to 94.38% by 72 h the highest final activity among all bacterial combinations. Sodium silicate plus CSX134 was right behind, reaching 90.88% at 72 h. Notably, only combinations containing CSX134 broke the 90% threshold, suggesting its metabolites play nicely with and may even boost the green chemicals.

The fungal combinations were equally promising, though their performance profiles varied (Table S5). Sodium silicate plus CSUFT-F23 maintained consistently high activity throughout, starting at 90.51% (24 h) and hitting 95.88% at 72 h. Arecoline plus CSZ71 was close behind, with 94.93% at 72 h. Some combos had a slow start but caught up fast—arecoline plus LYZ21, for instance, jumped from under 19% at 24 h to nearly 89% by 72 h. A few others, though, lagged behind and never reached 80%.

Based on these findings, five top-performing combinations were selected for further study (Table S6, Figure S2): arecoline plus CSX134, arecoline plus CSZ33, arecoline plus CSZ71, sodium silicate plus CSUFT-F23, and sodium silicate plus CSZ71. The microbial strains were identified as *Bacillus amyloliquefaciens* (CSX134, GenBank accession number: PP389390), *Penicillium sclerotiorum* (CSZ33, GenBank accession number: PP389403), *Arthropsis hispanica* (CSZ71, GenBank accession number: PP389402), and *Tolypocladium* sp. (CSUFT-F23, GenBank accession number: PV460607). Together with arecoline and sodium silicate, these will move forward into the next phase of experiments.

Collectively, these findings lay the groundwork for developing combined formulations, pairing the most promising microbial filtrates with these effective green substances to tackle pine wilt disease.

### 3.3. Green Compound Agent Induces Resistance to Pine Wood Nematode Disease in P. massoniana

#### 3.3.1. Changes in Ascorbate Peroxidase (APX) Activity Within *P. massoniana*

Ascorbate peroxidase (APX) is a frontline antioxidant enzyme in plants—it scavenges hydrogen peroxide, keeping oxidative damage in check and helping cells stay stable. The present work tracked APX activity in *P. massoniana* over 72 h (Figure S3). A consistent biphasic response was observed: an initial decline in activity was followed by a subsequent recovery.

Most combined treatments boosted APX levels above the control at multiple time points. Arecoline plus CSX134 outperformed single treatments at 24, 36, and 48 h, peaking at 36 h. Arecoline plus CSZ71 followed a similar arc, topping out at 36 h (1.80 U/g·min). Arecoline plus CSZ33 peaked later at 72 h (1.86 U/g·min), while arecoline plus CSUFT-F23 generally beat the fungal filtrate alone, though not always arecoline. Sodium silicate plus CSZ71 was the rock star, and it exceeded both individual treatments at every time point, peaking at 72 h (1.93 U/g·min). Sodium silicate plus CSUFT-F23 also outperformed single treatments, hitting its stride at 36 h. The bottom line is the following: while each combo had its own rhythm, nearly all of them enhanced APX activity beyond what either the microbial filtrate or the chemical agent could do alone.

**Figure 1:**
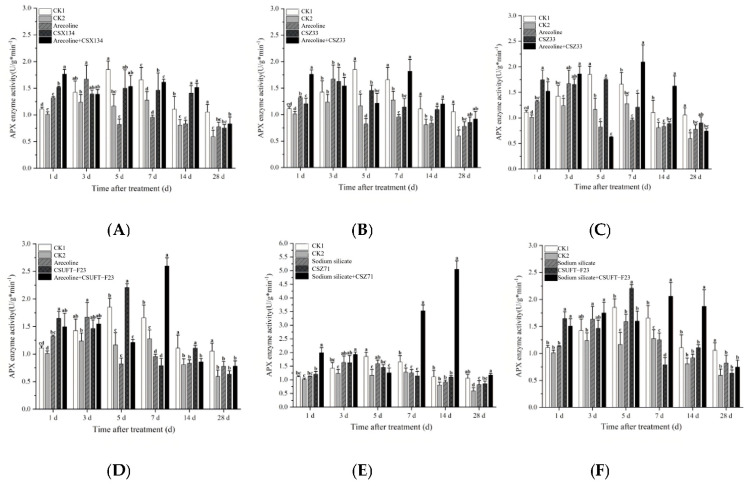
The changes in APX activity within *P. massoniana* seedlings under different treatments over 28 days. Note: (**A**): arecoline plus CSX134 composite treatment; (**B**): arecoline plus CSZ71 composite treatment; (**C**): arecoline plus CSZ33 composite treatment; (**D**): arecoline plus CSUFT-F23 composite treatment; (**E**): sodium silicate plus CSZ71 composite treatment; (**F**): sodium silicate plus CSUFT-F23 composite treatment; CK: treated with sterile water; CK1: treated with *B. xylophilus* control; lowercase letters indicate significant differences in the activity of APX in *P. massoniana* under different treatments at the same time point (means were compared with Duncan’s multiple range test at *p* < 0.05).

APX activity in *P. massoniana* seedlings was monitored over 28 days after inoculation with *B*. *xylophilus* and following root-drench treatments (Figure 1). Overall, the combined treatments consistently induced higher APX activity than the single-agent treatments in the later stages. In particular, the combinations arecoline plus CSZ33, arecoline plus CSUFT-F23, and sodium silicate plus CSZ71 showed notably stronger activity at several time points. Looking at the individual combinations, arecoline plus CSX134 showed a fluctuating pattern—activity rose and fell more than once. Although it remained above the single treatments from day 5 onward, its peak occurred early (1.76 U/g·min on day 1) and was not surpassed later, suggesting a relatively limited or short-lived induction effect. Arecoline plus CSZ71 activity declined initially before rising again, peaking at day 7 (1.817 U/g·min). At this point, it was about 1.9 times higher than arecoline alone and 1.6 times higher than the CSZ71 filtrate alone. Arecoline plus CSZ33 also exhibited a multi-phase trend, with a sharp peak at day 7 (2.09 U/g·min) 2.1 times the activity of arecoline alone and 1.7 times that of the CSZ33 filtrate alone. Arecoline plus CSUFT-F23 reached its highest activity (2.6 U/g·min) at day 7, after which it declined, indicating that the effect was most pronounced around one week after treatment. Sodium silicate plus CSZ71 maintained relatively high activity throughout, exceeding both individual treatments at every measured time point. The difference was especially clear on days 7 and 14. At day 14, activity reached 5.05 U/g·min, about 5.5 times that of sodium silicate alone and 4.6 times that of the CSZ71 filtrate alone. Sodium silicate plus CSUFT-F23 again displayed a fluctuating pattern, with noticeably higher activity than the single treatments on days 7 and 14. In summary, the combined treatments arecoline plus CSZ71, arecoline plus CSZ33, arecoline plus CSUFT-F23, and sodium silicate plus CSZ71 all significantly induced APX activity in pine seedlings. Among them, arecoline plus CSZ33, arecoline plus CSUFT-F23, and sodium silicate plus CSZ71 appeared particularly effective, producing stronger and more sustained induction over the 28-day period.

#### 3.3.2. Changes in Glutathione Reductase (GR) Activity in *P. massoniana*

Glutathione reductase (GR) is a key antioxidant enzyme that maintains cellular redox balance by recycling oxidized glutathione (GSSG) back to its reduced form (GSH). Under stress, GR activity serves as a reliable readout of how well cells are coping with oxidative damage.

GR activity in *P. massoniana* was monitored over a 72-h period after treatment (Figure S4). Overall, activity followed a rise dip rise pattern, but the details varied. Several combinations like arecoline plus CSZ71 peaked later (72 h), while others, such as arecoline plus CSUFT-F23, spiked early at 24 h (10.18 U/g·min) before tapering off. The sodium silicate combos also performed well, showing higher activity than single treatments at multiple time points. Most importantly, nearly all combined treatments boosted GR activity beyond single-agent controls, with peak activity clustered around 36–48 h. The different peak times across combinations hint that they may be activating defense pathways through slightly distinct mechanisms, some fast-acting, others more sustained.

**Figure 2:**
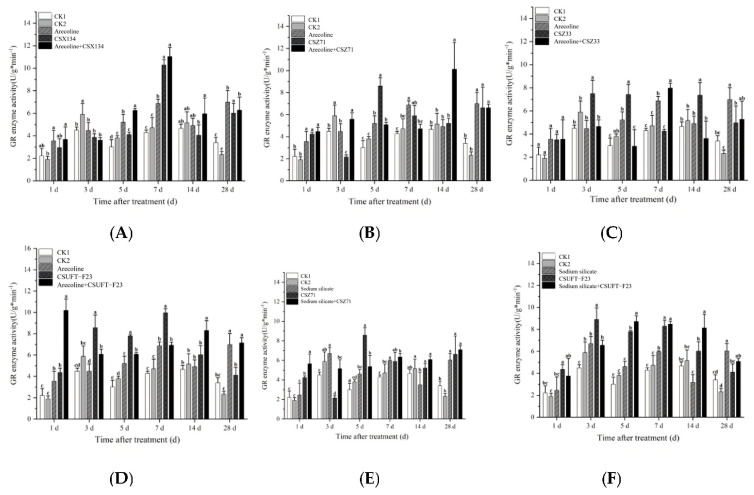
The changes in GR activity within *P. massoniana* seedlings under different treatments over 28 days. Note: (**A**): arecoline plus CSX134 composite treatment; (**B**): arecoline plus CSZ71 composite treatment; (**C**): arecoline plus CSZ33 composite treatment; (**D**): arecoline plus CSUFT-F23 composite treatment; (**E**): sodium silicate plus CSZ71 composite treatment; (**F**): sodium silicate plus CSUFT-F23 composite treatment; CK: treated with sterile water;CK1:treated with *B. xylophilus* control; lowercase letters indicate significant differences in the activity of GR in *P. massoniana* under different treatments at the same time point (means were compared with Duncan’s multiple range test at *p* < 0.05).

GR activity in *P*. *massoniana* seedlings was monitored over 28 days following inoculation with *B*. *xylophilus* (Figure 2). Data collected at 5, 7, 14, and 28 days after the initial 72 h treatment period are shown in Figure 2. Most combination treatments reached peak GR activity around days 7 and 14. Notably, arecoline plus CSX134, arecoline plus CSZ71, and sodium silicate plus CSZ71 showed significantly higher activity than their corresponding single-agent treatments at specific time points. Key trends for each combination: arecoline plus CSX134 activity rose, fell, and then rose again. It exceeded single treatments on days 5, 7, and 14, and remained higher than the filtrate-only group at day 28. Peak activity (11.04 U/g·min) occurred on day 7. Arecoline plus CSZ71 increased gradually before declining, peaking at day 14 (10.12 U/g·min). This was 2.06 times the activity of arecoline alone and 1.94 times that of the CSZ71 filtrate alone. Arecoline plus CSZ33 followed a down–up–down pattern. It was higher than the filtrate only treatment on days 1, 7, and 28, but the overall induction appeared somewhat limited in duration. Arecoline plus CSUFT-F23 declined initially before increasing, remaining above single treatments on days 1, 14, and 28 and suggesting a stable, longer lasting effect. Sodium silicate plus CSZ71 showed relatively steady activity, outperforming the filtrate-only treatment on days 1, 3, 14, and 28. The peak at day 14 (7.75 U/g·min) was 2.45 times that of silicate alone and 1.49 times that of the CSZ71 filtrate alone.

Activity in the sodium silicate plus CSUFT-F23 combination rose initially, then declined. Even so, it stayed clearly above the single treatments during the middle to late period. This difference was especially evident on days 5, 7, and 14. The highest value (8.71 U/g·min) was recorded on day 5. In summary, all tested combinations, arecoline plus CSX134, arecoline plus CSZ71, arecoline plus CSZ33, arecoline plus CSUFT-F23, sodium silicate plus CSZ71, and sodium silicate plus CSUFT-F23, induced GR activity in pine seedlings. Among them, arecoline plus CSZ71, sodium silicate plus CSZ71, and sodium silicate plus CSUFT-F23 appeared particularly effective, showing stronger and more sustained induction over the 28-day period.

#### 3.3.3. Changes in Polyphenol Oxidase (PPO) Activity in *P. massoniana*

Polyphenol oxidase (PPO) is something plants keep at the ready—it is stored in plastids and springs into action when they are wounded or infected. Once activated, it oxidizes phenolics into quinones, triggering tissue browning that helps seal off damage and fend off pathogens.

PPO activity in *P. massoniana* was tracked over 72 h (Figure S5). PPO levels went up, then dropped, and then rose again. This trend was largely consistent across treatments. Arecoline plus CSX134 peaked early at 24 h, then tapered off. Arecoline plus CSZ71 stayed strong across all time points, topping out at 214.25 U/g·min by 48 h. Arecoline plus CSZ33 hit an impressive 288.04 U/g·min at 36 h, the highest peak recorded. Arecoline plus CSUFT-F23 bounced around but peaked at 24 h, beating every single treatment at that moment. Both sodium silicate combos (plus CSZ71 and plus CSUFT-F23) outperformed single treatments at 12, 24, and 48 h.

Most combined treatments did more than simply match the single agents. They outlasted them and proved more potent. Each combo had its own pace and peak, but the trend was clear: pairing microbial filtrates with green chemicals gives PPO activity a real boost.

**Figure 3:**
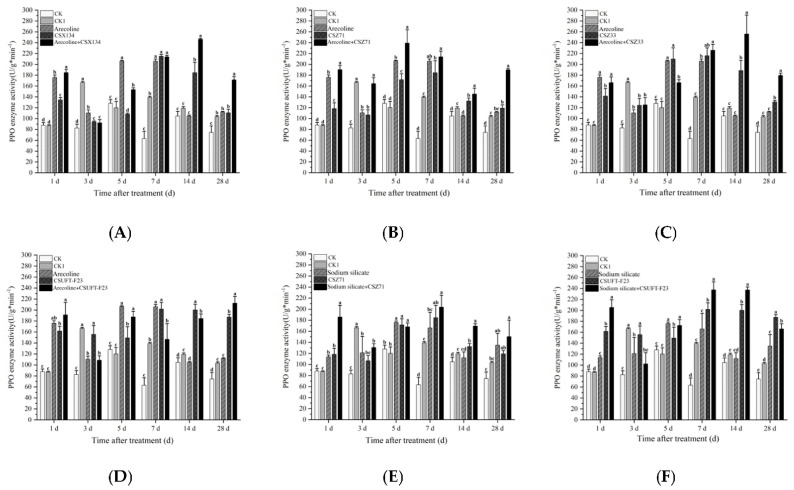
The changes in PPO activity within *P. massoniana* seedlings under different treatments over 28 days. Note: (**A**): arecoline plus CSX134 composite treatment; (**B**): arecoline plus CSZ71 composite treatment; (**C**): arecoline plus CSZ33 composite treatment; (**D**): arecoline plus CSUFT-F23 composite treatment; (**E**): sodium silicate plus CSZ71 composite treatment; (**F**): sodium silicate plus CSUFT-F23 composite treatment; CK: treated with sterile water;CK1:treated with *B. xylophilus* control; lowercase letters indicate significant differences in the activity of PPO in *P. massoniana* under different treatments at the same time point (means were compared with Duncan’s multiple range test at *p* < 0.05).

PPO activity in *P*. *massoniana* seedlings was tracked over 28 days after inoculation with *B*. *xylophilus* (Figure 3). In the later stages of the experiment, all combination treatments induced higher PPO activity than the corresponding single-agent treatments. Key results for each combination were as follows: Arecoline plus CSX134 showed an up–down–up pattern, with activity significantly exceeding single treatments at 14 and 28 days. The peak (246.02 U/g·min) was reached at day 14. Arecoline plus CSZ71 increased slowly before declining. The combined treatment consistently outperformed single treatments throughout, peaking at day 5 (239.14 U/g·min) and maintaining relatively stable activity at days 14 and 28. Arecoline plus CSZ33 followed a decrease increase decrease trend. During the mid to late period, it surpassed both single treatments, with a maximum of 256.03 U/g·min at day 14—2.45 times the activity of arecoline alone and 1.35 times that of the CSZ33 filtrate alone. Arecoline plus CSUFT-F23 decreased initially before rising, reaching its highest level (212.47 U/g·min) at day 28 while remaining above the single treatments. Sodium silicate plus CSZ71 exhibited a down up down pattern. Activity was higher than either single treatment during the mid to late phase, peaking at day 7 (203.58 U/g·min) and still exceeding single treatments at day 28. Sodium silicate plus CSUFT-F23 showed notably higher activity than single treatments on days 7 and 14, with the highest value (237.36 U/g·min) occurring on day 5. In summary, all tested combinations, arecoline plus CSX134, arecoline plus CSZ71, arecoline plus CSZ33, arecoline plus CSUFT-F23, sodium silicate plus CSZ71, and sodium silicate plus CSUFT-F23, effectively induced PPO activity in pine seedlings. Among them, arecoline plus CSX134, arecoline plus CSZ33, and sodium silicate plus CSUFT-F23 appeared particularly potent, producing stronger and more sustained induction over the 28-day period.

#### 3.3.4. Changes in Malondialdehyde (MDA) Content in *P. massoniana*

**Figure 4:**
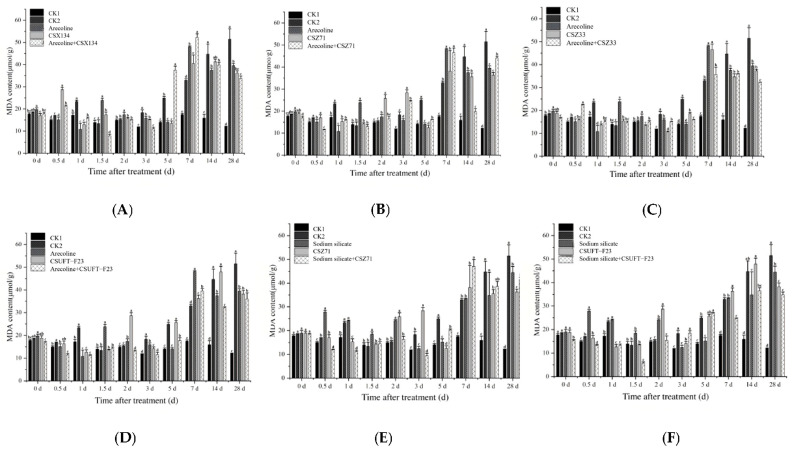
The changes in MDA content within the tissues of *P. massoniana* seedlings after different treatments over 28 days. Note: (**A**): arecoline plus CSX134 composite treatment; (**B**): arecoline plus CSZ71 composite treatment; (**C**): arecoline plus CSZ33 composite treatment; (**D**): arecoline plus CSUFT-F23 composite treatment; (**E**): sodium silicate plus CSZ71 composite treatment; (**F**): sodium silicate plus CSUFT-F23 composite treatment; CK: treated with sterile water; CK1:treated with *B. xylophilus* control; lowercase letters indicate significant differences in MDA content in *P. massoniana* between treatments at the same time point (means were compared with Duncan’s multiple range test at *p* < 0.05).

Malondialdehyde (MDA) is a product of membrane lipid peroxidation, generated in organisms during senescence or under stress. Accumulation correlates with aging severity. It also tracks closely with damage from both abiotic and biotic stress. Changes in MDA content in *P*. *massoniana* seedlings over 28 days are shown in Figure 4. Arecoline plus CSX134: MDA levels remained relatively stable during the first 3 days, peaked at 7 days after nematode inoculation, and then gradually declined. In the later stages, MDA content was lower than in the single treatments. Arecoline plus CSZ71: content rose slightly within the first 3 days, then stabilized, reaching its highest point at day 7. Arecoline plus CSZ33: over 28 days, MDA increased modestly by day 1.5, remained relatively steady afterward, and peaked at day 7 though at a level lower than the single treatments. By day 28, content was 19.21 μmol/g lower than the control. Arecoline plus CSUFT-F23: MDA rose gradually before decreasing. Throughout the early to middle period, content was generally lower than in single treatments, peaking at day 7 then declining. Sodium silicate plus CSZ71: MDA fluctuated within the first 3 days, increased gradually by day 5, and dropped again by day 14. Sodium silicate plus CSUFT-F23: content rose with some fluctuation over 28 days but remained mostly below other treatments, ending 16.61 μmol/g lower than the control at day 28. In summary, all tested combinations, arecoline plus CSX134, arecoline plus CSZ71, arecoline plus CSZ33, arecoline plus CSUFT-F23, sodium silicate plus CSZ71, and sodium silicate plus CSUFT-F23, contributed to reduced MDA accumulation in pine seedlings. Among them, arecoline plus CSZ33 and sodium silicate plus CSUFT-F23 appeared particularly effective, showing stronger and more sustained mitigation over the 28 day period.

#### 3.3.5. The Efficacy of Green Kill Line Compound Agent in Controlling Pine Wood Nematode Disease in *P. massoniana*

In terms of actual disease control (Table S7), all combinations provided some level of protection, with control efficacy rates as follows: arecoline plus CSX134: 46.37%; arecoline plus CSZ71: 47.82%; arecoline plus CSZ33: 76.81%; arecoline plus CSUFT-F23: 49.76%. Sodium silicate plus CSZ71: 37.20%; sodium silicate plus CSUFT-F23: 72.95%. Given their superior performance in both inducing plant resistance and achieving higher control efficacy, the combinations arecoline plus CSZ33 and sodium silicate plus CSUFT-F23 were selected for subsequent investigation into their effects on the expression of disease resistance related genes in pine seedlings.

**Figure 5:**
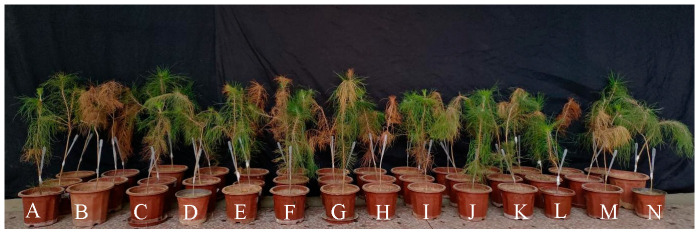
Morbidity of ponytail pine under different compounding agent treatments. Note: (**A**): CK: sterile water control; (**B**): CK1 pine nematode; (**C**): sodium silicate; (**D**): arecoline; (**E**): CSX134; (**F**): CSZ71; (**G**): CSUFT-F23; (**H**): CSZ23; (**I**): arecoline plus CSX134; (**J**): arecoline plus CSZ71; (**K**): arecoline plus CSZ33; (**L**): arecoline plus CSUFT-F23; (**M**): sodium silicate plus CSZ71; (**N**): sodium silicate plus CSUFT-F23.

Based on the changes in three defense-related enzyme activities and MDA content in *P. massoniana* over 28 days of treatment with various combinations, most combinations exhibited some level of resistance-inducing capacity. The control efficacy of these treatments against *B. xylophilus* at 28 days is shown in Figure 5. The results indicate that two combinations, arecoline plus CSZ33 and sodium silicate plus CSUFT-F23, were particularly effective. They not only showed direct nematicidal activity but also consistently induced stronger defense responses in the seedlings.

#### 3.3.6. Effects of Green Kill Line Compound Agent on the Expression Levels of Relevant Defense Genes in *P. massoniana*

**Figure 6:**
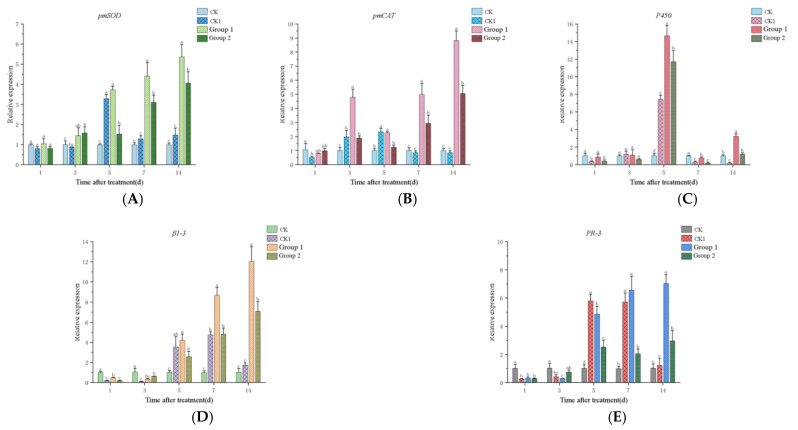
Expression of needle resistance genes in *P. massoniana* under different treatments (group 1: arecoline plus CSZ33; group 2: sodium silicate plus CSUFT-f23). Note: (**A**): *pmSOD*; (**B**): *pmCAT*; (**C**): cytochrome *P450*; (**D**): *PR-2* (β-1,3-glucanase); (**E**): *PR-3* (class l chitinase) (means were compared with Duncan’s multiple range test at *p* < 0.05). lowercase letters indicate significant differences in MDA content in *P. massoniana* between treatments at the same time point (means were compared with Duncan’s multiple range test at *p* < 0.05).

Following treatment with the selected combinations arecoline plus CSZ33 and sodium silicate plus CSUFT-F23. The expression of five defense- and stress-related genes in *P. massoniana* was analyzed: *PmSOD*, *PmCAT*, *cytochrome P450*, *PR-2* (β-1,3-glucanase), and *PR-3* (class I chitinase). Overall, gene expression generally increased over the 14-day treatment period for both combinations, though the patterns and magnitudes differed (Figure 6). Looking at the specifics: *PmSOD* (Figure 6A): expression increased steadily in both treatments. Arecoline plus CSZ33 induced a strong late-stage response, peaking at day 14 with levels 5.36 times higher than CK and 3.62 times higher than CK1. Sodium silicate plus CSUFT-F23 also peaked at day 14, though at a lower level than the arecoline combination. *PmCAT* (Figure 6B): expression followed a rise–fall–rise pattern. Arecoline plus CSZ33 induced a sharp peak at day 14 (8.75 times CK; 10.17 times CK1). Sodium silicate plus CSUFT-F23 showed a similar trend but with lower expression overall. Cytochrome *P450* (Figure 6C): expression generally rose and then fell. Arecoline plus CSZ33 produced the highest levels at days 5 and 14. Sodium silicate plus CSUFT-F23 peaked sharply at day 5, reaching 11.43 times the CK level. *PR-2*/β-1,3-glucanase (Figure 6D): expression increased gradually. Arecoline plus CSZ33 showed the highest levels in the mid-to-late period, peaking at day 14 at 11.59 times CK. Sodium silicate plus CSUFT-F23 also peaked at day 14, albeit at about half the level (6.81 times CK). *PR-3*/class I chitinase (Figure 6E): expression rose progressively. Arecoline plus CSZ33 again induced the strongest response by day 14 (6.82 times CK). Sodium silicate plus CSUFT-F23 expression, while lower earlier, still peaked above controls at day 14 (2.88 times CK). In short, both green combination treatments significantly upregulated the expression of key defense-related genes in pine seedlings over time. The arecoline plus CSZ33 combination consistently induced stronger expression across most genes and time points, while sodium silicate plus CSUFT-F23 also showed clear, though often milder, induction. These results indicate that the selected combinations can activate systemic defense pathways at the transcriptional level.

### 3.4. Metabolic Product Analysis of Fermentation Filtrates from Four Bacillus subtilis Strains

#### 3.4.1. Multivariate Statistical Analysis of Fermentation Filtrate

**Figure 7:**
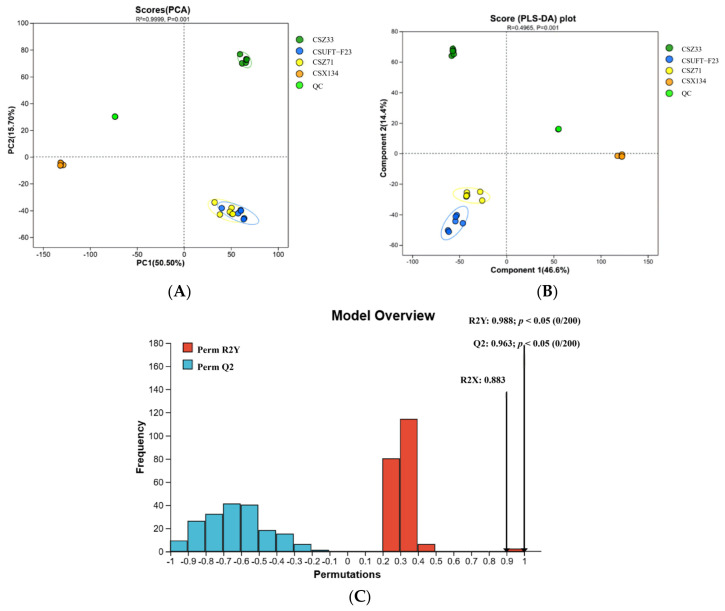
Multivariate statistical analysis plot of metabolites. Note: (**A**): plot of PCA scores of metabolites in fermentation filtrates of different strains of bacteria. (**B**): PLS-DA score plot of metabolites in the fermentation filtrate of the strain. (**C**): validation diagram of PLS-DA replacement model.

Mass spectrometry was used to obtain metabolic profiles from the fermentation filtrates. Four selected strains were analyzed. The bacterial strain CSX134 and the fungal strains CSZ33, CSZ71, and CSUFT-F23. The PCA score plot (Figure 7A) shows that all sample replicates fall within the confidence interval. Replicates from the same group cluster closely together, indicating good reproducibility and minimal within-group variation. Between groups, separation is evident along principal component 1 (PC1). Notably, the clusters for CSZ71 and CSUFT-F23 are positioned near each other, suggesting a degree of similarity in their metabolic profiles. PC1 explains 50.50% of the total variance, while PC2 explains 15.70%, collectively accounting for 66.20% of the variance a result that supports reliable pattern recognition. The PLS-DA score plot (Figure 7B) reveals a clearer separation between groups. The bacterial filtrate (CSX134) is distinctly separated from the three fungal groups, highlighting a significant metabolic difference between bacterial and fungal fermentation products. CSZ33 also shows a distinct profile. As suggested by the PCA, CSZ71 and CSUFT-F23 remain closer to each other, though subtle differences are present. These results demonstrate clear separation trends between sample groups and tight clustering of replicates, confirming the reliability of the data processing. Model validation metrics (Figure 7C) further support the robustness of the PLS-DA model. Here, R2X (0.883) represents the explained variance in the metabolic data (X-matrix), R2Y (0.988) indicates the model’s explanation of the class assignment (Y-matrix), and Q2 (0.963) reflects its predictive capability. A Q2 value closer to 1 denotes a stronger model. The obtained high values, along with significant *p*-values for Q2 and R2Y (*p* < 0.05), confirm that the model is valid and effective, providing a solid foundation for subsequent data analysis.

**Figure 8:**
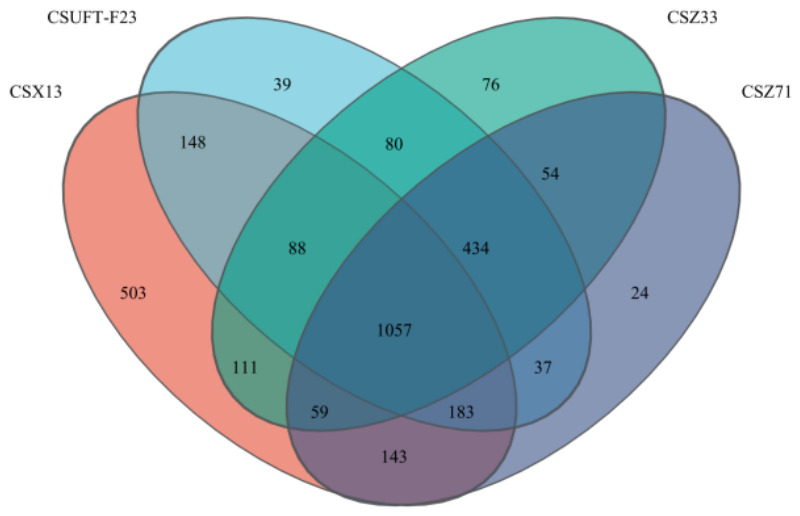
Venn diagram of metabolites in fermentation filtrate of different strains.

Based on mass spectrometry analysis, 1057 metabolites were found to be common across the fermentation filtrates of the four selected strains (Figure 8). The number of unique metabolites, however, varied considerably among them. The bacterial filtrate (CSX134) contained the highest number of unique metabolites (503), including compounds such as monocrotaline, beta-acetyldigoxin, epothilone C, acetophenazine, and threonic acid. In contrast, the fungal filtrates had far fewer unique metabolites: 39 for CSUFT-F23 (e.g., fiscalin C, linsitinib), 76 for CSZ33 (e.g., 3′-hydroxybuspirone, glucosamine 6-sulfate), and only 24 for CSZ71 (e.g., phaseol, 7-methylinosine). This substantial difference, particularly the high number of unique metabolites in the bacterial filtrate, strongly suggests that the metabolic profiles of bacterial and fungal fermentation products are distinctly different.

#### 3.4.2. Annotation Analysis of Co-Metabolites in Fermentation Filtrates

**Figure 9:**
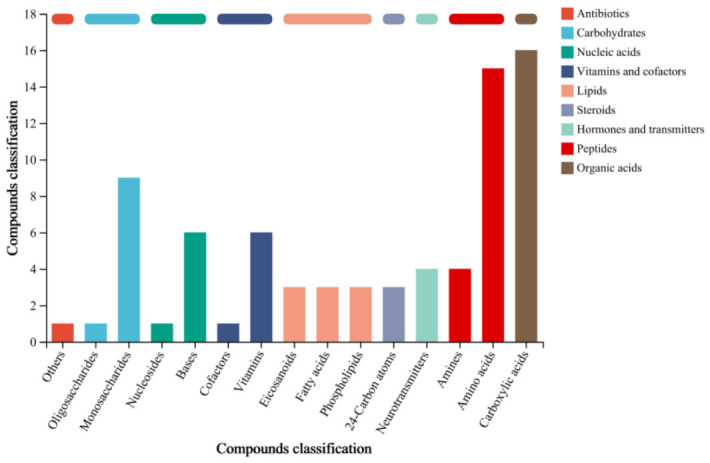
Classification statistics of common compounds in the fermentation filtrate of strains.

Metabolic profiling of the fermentation filtrates from the four strains bacterial CSX134 and fungal CSZ33, CSZ71, and CSUFT-F23 revealed their shared metabolites across major biological categories (Figure 9). The KEGG secondary classification is shown on the horizontal axis, with the number of metabolites in each category on the vertical axis. The identified metabolites were grouped into several functional classes: organic acids, lipids, carbohydrates, nucleic acids, peptides, vitamins and cofactors, steroids, hormones and transmitters, and antibiotics. Among the annotated metabolites, carboxylic acids (a subclass of organic acids) were the most abundant, with 16 compounds detected. These included acetoacetic acid, adipic acid, glutaric acid, citric acid, succinic acid, ketoleucine, and 3-methyl-2-oxovaleric acid. Next were amino acids and related peptides, totaling 15 metabolites such as L-methionine, pyroglutamic acid, GABA, L-threonine, L-tyrosine, 3-aminoisobutanoic acid, and L-dopa. Four amines were also identified. Monosaccharides and derivatives under the carbohydrate category numbered nine, with examples being muramic acid, N-acetylneuraminic acid, D-sorbitol, rhamnose, N-acetylmuramate, and D-erythrose. In addition, six vitamins and cofactors were detected, including niacinamide, pyridoxal, riboflavin (vitamin B2), and ascorbic acid.

**Figure 10:**
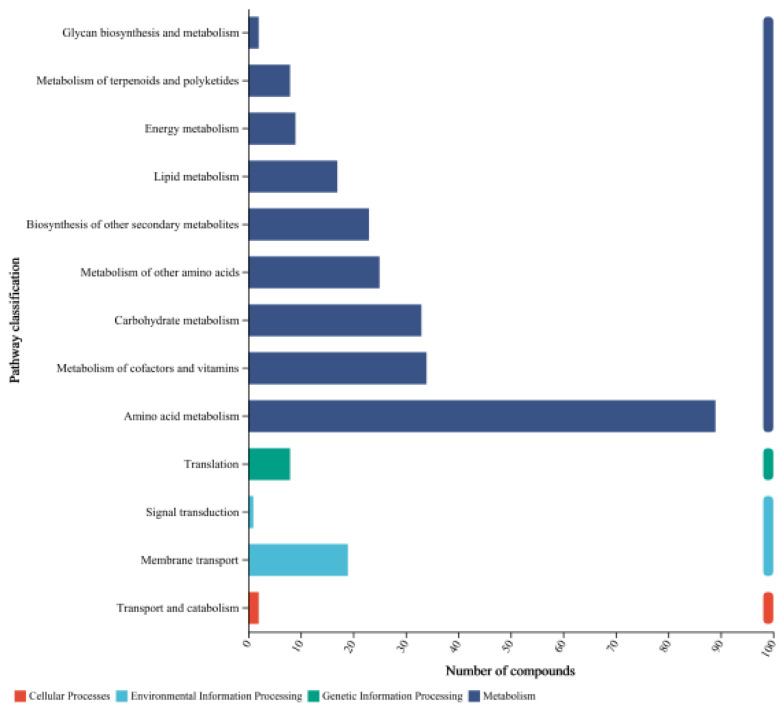
Analysis of KEGG pathway for common metabolites in fermentation filtrate of strains.

Metabolites shared across the fermentation filtrates of all four strains were first identified and then subjected to KEGG enrichment analysis. The results are shown in Figure 10, where the *y*-axis represents the secondary classification of KEGG pathways and the *x*-axis shows the number of metabolites annotated to each pathway. The analysis revealed that the most significantly enriched pathways among the shared metabolites were primarily related to metabolism. These included amino acid metabolism, metabolism of cofactors and vitamins, carbohydrate metabolism, biosynthesis of other secondary metabolites, lipid metabolism, metabolism of terpenoids and polyketides, and glycan biosynthesis and metabolism. Additionally, several pathways linked to genetic information processing, environmental information processing, and cellular processes were also enriched. Overall, this enrichment pattern highlights core metabolic functions and stress-responsive pathways that are likely active across the different microbial filtrates. Together, these four classes represented the most prominently detected groups of metabolites in the fermentation filtrates of all four strains.

**Figure 11:**
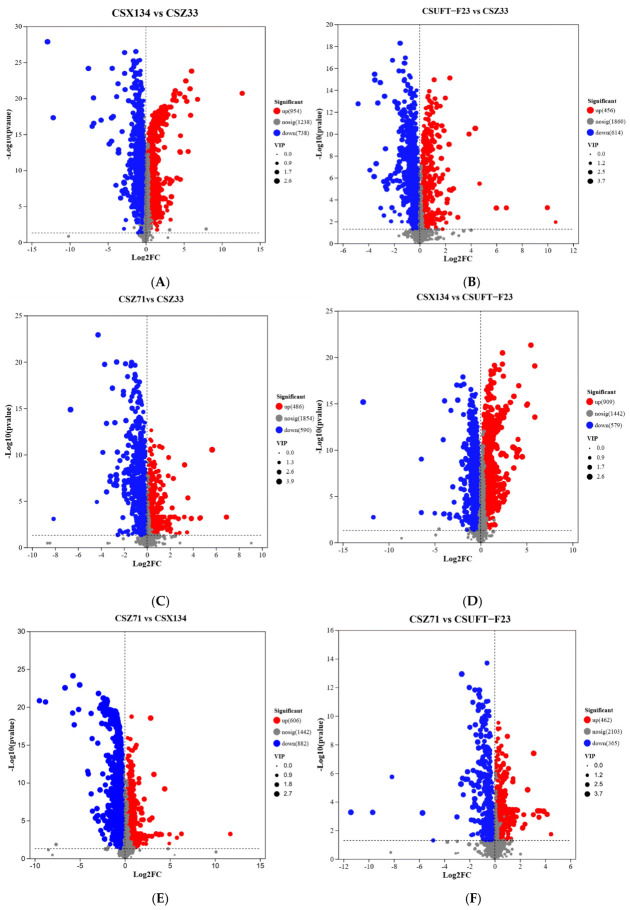
Screening of differential metabolites in fermentation filtrates of different strains. Note: (**A**): CSX134 vs. CSZ33; (**B**): CSUFT-F23 vs. CSZ33; (**C**): CSZ71 vs. CSZ33; (**D**): CSX134 vs. CSUFT-F23; (**E**): CSZ71 vs. CSX134; (**F**): CSZ71 vs. CSUFT-F23.

To explore the metabolic differences among the fermentation filtrates of the four selected strains, pairwise comparisons were performed to identify differentially abundant metabolites. The results are presented as volcano plots (Figure 11), where metabolites positioned further to the left/right and toward the top represent more significant changes. Each point corresponds to a specific metabolite, with point size indicating the VIP score. Red, blue, and gray points denote significantly upregulated, downregulated, and non-significant metabolites, respectively. Key findings from each comparison were as follows: CSX134 vs. CSZ33 (Figure 11A): 954 metabolites were significantly upregulated and 738 were downregulated. Notable upregulated compounds included blennin B, myotoxin A, norepinephrine, and hovenidulcioside B2, while ferreirin and cedazuridine were among the significantly downregulated ones. CSUFT-F23 vs. CSZ33 (Figure 11B): 456 metabolites were upregulated and 614 were downregulated. Upregulated examples included ximoprofen and benzocaine, whereas simulansamide and acetylphosphate were notably downregulated. CSZ71 vs. CSZ33 (Figure 11C): 486 metabolites were upregulated and 590 were downregulated. Upregulated metabolites featured 7 methylinosine and gibberellin A59, while phesergly and ferreirin were downregulated. CSX134 vs. CSUFT-F23 (Figure 11D): 909 metabolites were upregulated and 579 were down-regulated. Asp ile, leu phe, and pentostatin were upregulated; chitin and maltopentaose were among the downregulated compounds. CSZ71 vs. CSX134 (Figure 11E): 606 metabolites were upregulated and 882 were downregulated. Cedazuridine and isoscopoletin were upregulated, while methionyl lysine and 8 butanoylneosolaniol were downregulated. CSZ71 vs. CSUFT-F23 (Figure 11F): 462 metabolites were upregulated and 365 were downregulated. Glu leu gly and gabazine were upregulated, whereas colchicine, buntanine, and schidigerasaponin C2 were downregulated. In summary, pairwise comparisons revealed substantial differences in metabolite profiles between the bacterial and fungal filtrates, as well as among the fungal strains themselves. The bacterial filtrate (CSX134) showed the most distinct profile, consistent with its higher number of unique metabolites identified earlier.

#### 3.4.3. Verification of the Activity of Metabolites Against Pine Wood Nematode

Based on the metabolomics data of the microbial fermentation filtrates, nine commercially available metabolite standards were selected for further testing. A uniform concentration of 1 g/L was adopted for the nematicidal assays against *B. xylophilus*, a choice guided primarily by the need for practical comparability across different compounds. The results are summarized in Table 1. L-Ascorbic acid and niacinamide showed relatively low activity at 24 h, but by 72 h their mortality rates increased to 61.56% and 70.02%, respectively. While these findings are promising, the present study notes that establishing a direct, quantitative link between these exogenous concentrations and the endogenous levels within the rhizosphere will require targeted analytical work in future investigations. In contrast, D-glutamic acid and D-aspartic acid exhibited strong nematicidal effects early on, reaching corrected mortality rates of 94.95% and 94.13% at 72 h. These results confirm that specific metabolites present in the microbial fermentation filtrates possess direct nematicidal activity, supporting the role of metabolic products in the observed biocontrol effects.

## 4. Discussion

This study compares the distinct modes of action and temporal dynamics of bacterial versus fungal filtrates. A key finding was the divergent performance profiles of bacterial and fungal-based combinations. Combinations featuring the bacterial strain CSX134 (*B. amyloliquefaciens*) often exhibited a more gradual increase in nematicidal activity but induced strong and sustained defense enzyme responses (e.g., GR activity). This is similar to related reports [56,57]. In contrast, several fungal-based combinations, particularly sodium silicate plus CSUFT-F23, achieved very high early kill rates. This temporal difference likely stems from distinct metabolite profiles. Metabolomic analysis revealed that CSX134 possessed over 500 unique metabolites far more than any fungal strain tested. This metabolite pool is rich and complex. It contains compounds like certain alkaloids and organic acids. Against nematodes, it may act along several slower biochemical routes. At the same time, it seems to deliver a stronger, more persistent immune signal to the plant. The rapid action of some fungal combinations could be attributed to specific, potent secondary metabolites. This distinction underscores the importance of selecting microbial partners based on the desired outcome: rapid knockdown versus long-term system induction [58,59,60].

Activating multiple layers of defense is crucial for induced resistance. Data offer clear evidence on this point. The most effective combinations did not act solely as toxins. They also functioned as plant defense stimulants. A coordinated upregulation of antioxidant enzymes APX, GR, and PPO, along with defense-related genes, including *PmSOD*, *PmCAT*, *PR-2*, and *PR-3*, occurred. At the same time, malondialdehyde (MDA) levels decreased. This pattern fits a priming response rather than an immediate, constitutive activation. The timing tells us something important. The combined formulations triggered a rapid enzymatic antioxidant defense first, then reinforced it through upregulated gene expression. Compared to direct toxicity alone, this priming mechanism is more sustainable. It offers longer-lasting protection. This mechanism is more sustainable than direct toxicity alone, as it empowers the host plant, potentially leading to longer-lasting protection and reduced selection pressure for nematode resistance [61,62].

Metabolomic insights serve as a bridge connecting efficacy and mechanism. The non-targeted metabolomics analysis offers a plausible chemical basis for the observed bioactivities. The significant enrichment of pathways like amino acid metabolism and biosynthesis of other secondary metabolites in the shared metabolite pool highlights the metabolic potential of these strains for producing bioactive compounds. These results confirm that D-glutamic acid and D-aspartic acid possess direct nematicidal activity when tested as pure standards, suggesting that they may contribute to the overall activity of the fermentation filtrates, although their exact contribution in the complex mixture remains to be quantified. Absolute concentrations of D-glutamate and D-aspartate in the fermentation filtrates were not measured. Therefore, it cannot be concluded that these two compounds alone account for the observed nematicidal activity. Rather, they are considered members of a broader pool of bioactive metabolites that jointly drive the effect. Interestingly, while CSX134 had the most unique metabolites, the highly effective fungal strain CSZ33 (*P*. *sclerotiorum*) also shares a taxonomic link with fungi known for producing antimicrobial and anti-inflammatory compounds, supporting the idea that its efficacy may derive from specific, potent secondary metabolites. The metabolic divergence between CSX134 and the fungal strains, clearly shown in the PCA/PLS-DA plots, provides a foundational explanation for their different performance and enhanced efficacy when combined with chemical agents [63,64].

In conclusion, this study validates a strategy of creating hybrid bio-chemical formulations for managing pine wilt disease. The most promising combinations, such as arecoline plus CSZ33 and sodium silicate plus CSUFT-F23, successfully merge direct nematicidal action with the induction of a broad-spectrum plant immune response. The differences in efficacy and timing between bacterial and fungal-derived formulations enrich the toolkit available for integrated pest management, allowing for tailored solutions [65,66]. It should be noted that the present study used a single concentration (1.5 g/L) and a fixed 1:1 volumetric ratio for all combinations. While this concentration was selected based on preliminary dose–response data for the individual agents, factorial designs testing multiple ratios would be necessary to fully capture potential enhanced efficacy or antagonism. Results offer a proof of concept. The combinations tested led to measurably better control. That points to a clear need for further optimization. Systematic formulation development is the logical next step toward practical application. That work would include shelf-life stability, surfactant addition, and appropriate delivery devices.

This moves beyond a purely phenomenological observation (i.e., “this combination works”) toward a mechanistic understanding (i.e., “it works due to the presence and enhanced efficacy of specific classes of metabolites”). The broader implication of this work is that it validates a pragmatic and scalable model for integrated pest management. In an era of increasing pesticide resistance and environmental concern, strategies that reduce reliance on single high-dose chemical applications are critical. Using lower doses of green chemicals enhanced by biological agents offers a viable path forward. It aligns with the global shift toward sustainable agriculture, where solutions must be effective, environmentally sound, and economically feasible. Looking ahead, several key steps are needed to translate this proof of concept into practical tools. First, field trials are essential to confirm efficacy under real world conditions and to assess environmental fate. Second, formulation science must optimize the ratios, stability, and delivery methods for these combinations. Third, the molecular mechanism of induced resistance warrants deeper investigation using transcriptomic and proteomic tools to map the activated signaling pathways (e.g., jasmonic acid or phenylpropanoid biosynthesis). Finally, the fermentation processes for the promising microbial strains should be optimized to maximize the yield of the active metabolite fractions identified in this study. In summary, this research provides a compelling case for hybrid biochemical solutions against plant parasitic nematodes. By successfully integrating direct toxicity with plant immunity induction, a resilient and sustainable defense strategy is outlined. The journey from the laboratory bench to the forest will require further work, but the foundational principle that enhanced efficacy between biology and chemistry can yield superior, sustainable outcomes is clearly established.

## 5. Conclusions

This study demonstrates that combining microbial fermentation filtrates with green chemical agents creates a powerful, dual-action strategy for managing pine wilt disease. The most effective formulations, particularly arecoline plus CSZ33 and sodium silicate plus CSUFT-F23, achieved high direct nematicidal activity (>72% control) while simultaneously priming the innate immune system of *P*. *massoniana* seedlings. This dual approach, which merges immediate pathogen suppression with long-term host resistance, represents a meaningful advance toward sustainable pine wilt disease management. The core innovation lies in the observed enhanced efficacy. The combinations are not simply adding two effects together; they often performed better than either component alone. For instance, specific formulations boosted defense enzyme activities (APX, GR, PPO) several-fold compared to single treatments and significantly reduced oxidative damage (MDA content). Enhanced efficacy likely arises from complementary mechanisms: the chemical agents may disrupt nematode integrity or physiology, while the complex metabolite cocktails in the microbial filtrates both attack the pathogen and trigger broad-spectrum plant defense responses. Bacterial and fungal partners did not perform alike. *B. amyloliquefaciens* CSX134 was tested as the bacterial representative, with *P. sclerotiorum* CSZ33 and *Tolypocladium* sp. CSUFT-F23 as the fungal representatives. Bacterial combinations sometimes showed a slower onset but produced more sustained effects. This contrast clearly tells us one thing: the biological component is not interchangeable. The choice of microbial partner dictates the mechanistic profile and timing of the integrated response. Metabolomics provided a crucial link between efficacy and mechanism. The analysis confirmed that bacterial and fungal filtrates possess distinctly different metabolic profiles, with the bacterial strain CSX134 producing a particularly rich array of unique metabolites. Identifying nematicidal compounds like D-glutamic acid and D-aspartic acid within these profiles directly connects the chemical composition of the filtrates to their biological function.

## Figures and Tables

**Table 1:** Determination of metabolite nematicidal activity.

Name	24 h Corrected Mortality Rate (%)	48 h Corrected Mortality Rate (%)	72 h Corrected Mortality Rate (%)
Gibberellic acid	10.05 ± 1.30 cd	23.69 ± 5.22 e	23.85 ± 2.91 de
Sorbitol	27.00 ± 7.72 b	38.11 ± 7.83 d	57.58 ± 5.13 b
D-glutamic acid	57.94 ± 3.82 a	91.67 ± 4.11 a	94.95 ± 4.46 a
D-aspartic acid	60.91 ± 7.29 a	92.95 ± 1.76 a	94.13 ± 2.40 a
D-Serine	4.52 ± 3.08 d	12.30 ± 4.32 f	34.60 ± 4.31 cd
L-ascorbic acid	15.57 ± 9.03 c	67.76 ± 5.19 b	61.56 ± 9.33 b
Rhamnose	9.60 ± 0.93 cd	17.45 ± 4.88 ef	39.75 ± 10.94 c
Nicotinamide	5.91 ± 3.80 d	54.65 ± 1.62 c	70.02 ± 2.62 c
2′-Deoxycytidine	7.08 ± 1.80 d	11.69 ± 5.66 f	17.86 ± 4.81 e

Note: Different lower case letters in the same column indicate significant differences in the nematicidal activity of each metabolite at a uniform treatment time (means were compared with Duncan’s multiple range test at *p* < 0.05).

## Data Availability

The original contributions presented in the study are included in the article; further inquiries can be directed to the corresponding author.

## Supplementary Materials

The following supporting information can be downloaded at: https://www.mdpi.com/article/10.3390/microorganisms14061202/s1, Figure S1: The activity of different concentrations of green chemicals against *B.xylophilus*; Figure S2. ITS-rRNA phylogenetic tree. Note: (A): CSZ33; (B): CSUFT-F23; (C): CSZ71; (D): CSX134. Figure S3. The changes in APX activity within *Pinus massoniana* seedlings under different treatments over 72 h. Figure S4. The changes in GR activity within *P. massoniana* seedlings under different treatments over 72 h. Figure S5. The changes in PPO activity within *P. massoniana* seedlings under different treatments over 72 h. Table S1: Bacterial fermentation filtrate against *Bursaphelenchus xylophilus*. Table S2. Fungal fermentation filtrate against *B.xylophilus*. Table S3. Determination of the activity of 1.5 g/L green agent to kill *B.xylophilus*. Table S4. Nematicidal activity of Bacterial fermentation filtrate compounded with Green substances. Table S5. Nematicidal activity of Fungal fermentation filtrate compounded with Green substances. Table S6. Compound combination kill rate. Table S7. Effect of different treatments on the control of pine wilt disease in *P. massoniana*. Table S8. Related media formulations. Table S9. Criteria for grading *B. xylophilus* diseases. Table S10. Sequences of qPCR primers for mRNAs.

## Abbreviations

The following abbreviations are used in this manuscript:

APX	Ascorbate peroxidase
GR	Glutathione reductase
PPO	Polyphenol oxidase
MDA	Malondialdehyde
SOD	Superoxide dismutase
CAT	Catalase
PAL	Phenylalanine ammonialyase
*PmSOD*	Superoxide dismutase gene
*PmCAT*	Catalasegene gene
*P**mPOD*	Proso millet peroxidase gene
*P450*	Cytochrome monooxygenase gene
*PR-2*	β-1,3-glucanase gene
*PR-3*	Class l chitinase gene
