# Supplementary material for: Enhanced Efficacy of Rhizosphere Microorganisms and Green Compounds: A Dual-Action Strategy Against Bursaphelenchus xylophilus in Pinus massoniana
Source: Microorganisms. 2026 May 26;14(6):1202. doi: 10.3390/microorganisms14061202 (PMC13303170; doi:10.3390/microorganisms14061202)
Supplement: Supplementary file 1 [file microorganisms-14-01202-s001.zip › Supplementary materials file S1. Supplementary figures and tables.pdf]

Enhanced Efficacy of Rhizosphere Microorganisms and Green Compounds: A Dual-action Strategy Against *Bursaphelenchus xylophilus* in *Pinus massoniana*

Jiacheng Zhu[1], Yi Dang[2], Xiaoming Ren [3],Long Xu[3] ,Yilong Zhou[4] ,Guoying Zhou [1,2,3,4*], and Junang Liu [1,2,3,4*]

1 College of Forestry, Central South University of Forestry and Technology, Changsha 410004, China
2 Hunan Provincial Key Laboratory for Control of Forest Diseases and Pestsand Pests in South China, Central South University of Forestry and Technology, Changsha 410004, China
3 Key Laboratory for Non-Wood Forest Cultivation and Conservation of Ministry ofEducation, Central South University of Forestry and Technology, Changsha 410004, China
4 Yuelushan Laboratory Non-wood Forests Variety Innovation Center, Central South University of Forestry and Technology, Changsha 410004, China
* Correspondence: zgyingqq@163.com (G.Z.); kjc9620@163.com (J.L.)

Table S1. Bacterial fermentation filtrate against *Bursaphelenchus xylophilus.*

Strain No	24h corrected mortality rate (%)	48h corrected mortality rate (%)	72h corrected mortality rate (%)
CSUFT-B13	42.04±7.74def	67.38±5.02efg	77.55±1.41de
CSUFT-B15	63.54±4.09c	71.82±1.47cdef	75.98±2.76ef
CSX30	78.18±7.74ab	81.45±2.28ab	91.05±3.95ab
CSUFT-B33	36.11±1.52ef	67.04±3.41fg	71.74±4.97fg
CSUFT-B49	76.97±2.09ab	79.66±2.97abc	82.14±1.52cd
CSUFT-B54	46.93±15.21de	57.14±7.75h	87.00±0.81bc
CSUFT-B56	41.17±5.24def	76.29±4.31bcd	82.37±1.71cd
CSUFT-B60	48.94±8.21d	67.96±3.21defg	70.61±3.82g
CSUFT-B63	30.73±5.65f	61.20±12.72gh	73.49±4.45efg
CSUFT-B67	46.49±11.50de	75.14±3.29bcdef	82.66±1.48c
LYX73	72.60±3.27bc	75.85±4.95bcde	86.98±5.56bc
CSX134	85.90±3.42a	86.69±1.18a	93.06±2.03a

Note: Different lower case letters in the same column indicate significant differences in nematicidal activity between treatments (means were compared by Duncan's multiple range test at $p < 0.05$)

Table S2. Fungal fermentation filtrate against *B.xylophilus.*

Strain No	24h corrected mortality rate (%)	48h corrected mortality rate (%)	72h corrected mortality rate (%)
CSUFT-F6	48.17±8.43d	71.07±3.74c	80.62±4.37de
CSUFT-F7	69.19±2.94b	84.81±0.96ab	86.18±3.83cd
CSUFT-F19	55.41±4.61c	69.74±3.90c	71.13±6.03f
LYZ21	69.94±8.01b	71.10±12.34c	88.98±3.04bc
CSUFT-F23	74.29±2.58b	81.70±4.48b	90.85±0.66bc
CSUFT-F32	70.50±0.53b	84.97±2.39ab	88.62±0.76bc
CSZ33	82.78±4.06a	88.27±0.17ab	93.70±3.76b
CSUFT-F45	48.13±5.27d	61.19±3.04d	81.46±3.05de
CSUFT-F48	73.72±0.81b	81.14±1.55b	82.61±1.98de
CSUFT-F52	82.72±4.99a	81.63±5.47b	78.52±6.18e
CSUFT-F57	26.09±2.80e	69.55±3.94c	82.60±4.50de
CSZ71	85.79±0.82a	91.26±2.54a	94.55±1.37b

Note: Different lower case letters in the same column indicate significant differences in nematicidal activity between treatments (means were compared by Duncan's multiple range test at $p < 0.05$)

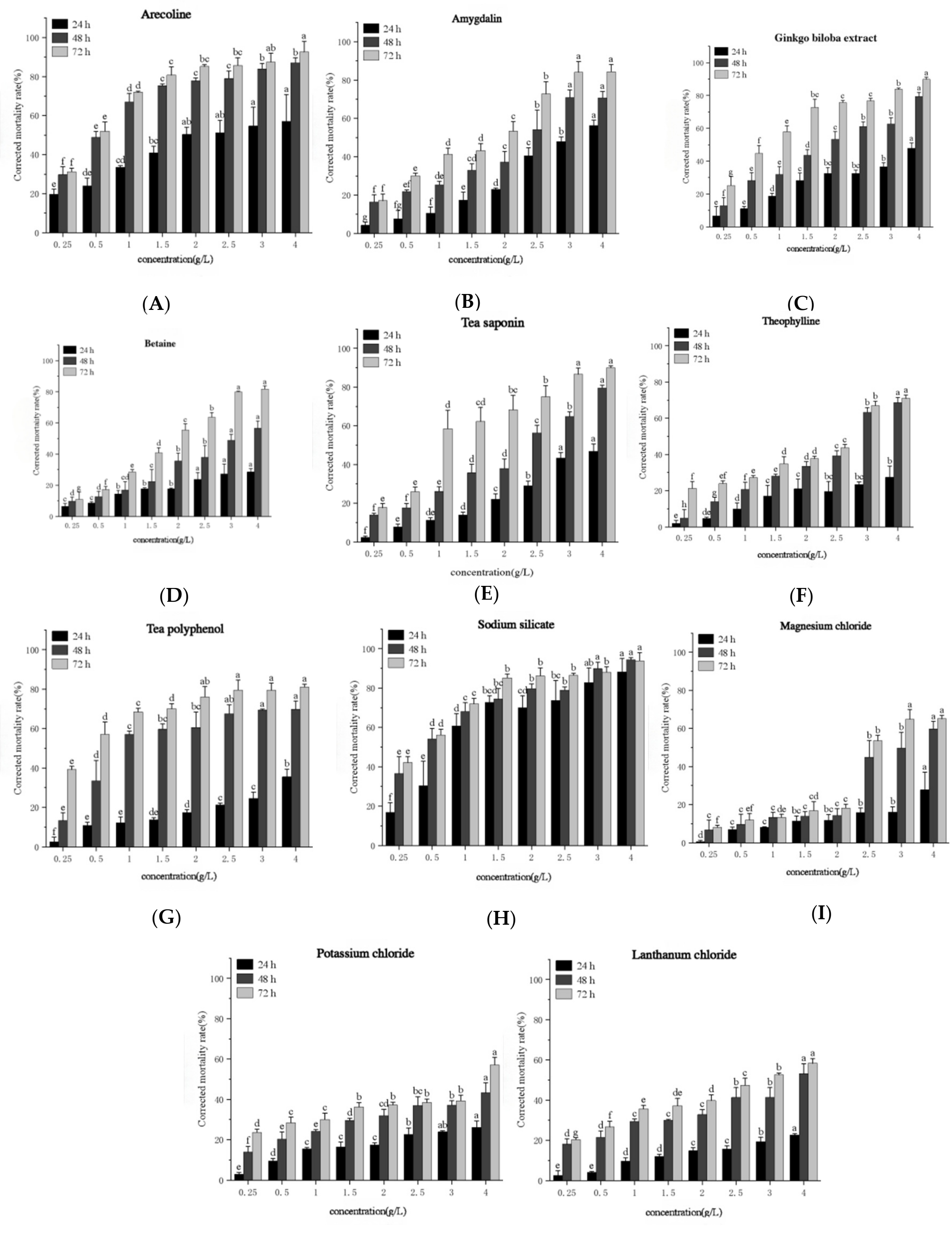

Figure S1. The activity of different concentrations of green chemicals against *B.xylophilus.* Note: (**A**): arecoline treatment; (**B**): amygdalin treatment; (**C**): ginkgo biloba extract treatment; (**D**): betaine treatment; (**E**): tea saponin treatment; (**F**): theophylline treatment; (**G**): tea polyphenol treatment; (**H**): sodium silicate treatment; (**I**): magnesium chloride treatment; (**J**): potassium chloride treatment; (**K**): lanthanum chloride treatment; lower case letters mean that the different treatments differed significantly in their pine nematode-killing activity during the same time period (means were compared by Duncan's multiple range test at $p < 0.05$)

Table S3. Determination of the activity of 1.5 g/L green agent to kill *B.xylophilus.*

Name	24h corrected mortality rate (%)	48h corrected mortality rate (%)	72h corrected mortality rate (%)
Sodium silicate 1.5g/L	72.73±3.30a	74.42±5.39a	85.16±1.88a
Arecoline 1.5g/L	40.90±3.46b	75.29±0.99a	80.75±4.26a
Ginkgo biloba extract 1.5g/L	28.27±4.49c	43.68±3.30c	72.70±5.10b
Amygdalin 1.5g/L	17.45±4.18d	33.02±3.34de	43.22±3.63d
Betaine 1.5g/L	17.78±0.63d	22.42±7.73f	40.81±3.23de
Theobromine 1.5g/L	13.85±1.53def	35.71±4.33d	62.24±7.22c
Theophylline 1.5g/L	17.12±5.82d	28.08±1.05e	34.85±4.01e
Tea polyphenols 1.5g/L	13.78±1.01def	59.65±2.67b	70.02±2.62b
Magnesium chloride 1.5g/L	11.47±2.59f	13.97±2.44g	16.90±4.74f
Potassium chloride 1.5g/L	16.40±2.50de	29.57±1.18e	36.22±2.25e
Lanthanum chloride 1.5g/L	12.08±0.99ef	29.97±0.56e	37.19±3.70de

Note: Different lower case letters in the same column mean that there is a significant difference between the nematicidal activity of different nematicides in the same treatment time. (means were compared by Duncan's multiple range test at $p < 0.05$)

Table S4. Nematicidal activity of Bacterial fermentation filtrate compounded with Green substances.

Compounding Agent		24h corrected mortality rate (%)	48h corrected mortality rate (%)	72h corrected mortality rate (%)
1.5g/L Arecoline	CSX134	69.89±2.63a	85.92±4.43a	94.38±0.61a
	CSX30	39.07±1.72gh	73.28±4.26efg	81.34±3.46fgh
	CSUFT-B54	64.43±4.18b	71.96±2.047efgh	77.76±4.63hi
	CSUFT-B56	40.60±2.58gh	79.38±3.09bc	84.74±1.17def
	CSUFT-B67	37.53±3.06h	56.61±2.79k	79.28±1.15ghi
	LYX73	53.63±3.31ef	68.72±2.91hij	86.42±3.17bcd
1.5g/L Sodium silicate	CSX134	56.19±1.07de	81.66±1.97ab	85.22±3.88def
	CSX30	64.04±3.53b	70.84±2.10fghi	81.94±4.36efg
	CSUFT-B54	42.44±3.64g	70.36±2.35ghi	82.20±2.50efg
	CSUFT-B56	49.18±3.01f	81.27±3.02ab	88.20±2.50bc
	CSUFT-B67	59.19±4.80cd	69.58±3.10ghi	77.08±3.14i
	LYX73	62.17±2.30bc	77.23±3.87bcd	82.97±3.35ef
1.5g/L Ginkgo biloba e xtract	CSX134	73.72±2.25a	84.11±1.99a	90.88±0.32ab
	CSX30	57.37±4.31de	73.54±4.33defg	85.89±3.93cde
	CSUFT-B54	39.55±3.55gh	66.82±2.54ij	88.02±1.61bc
	CSUFT-B56	57.81±2.24cde	64.36±3.02j	86.56±4.87bcd
	CSUFT-B67	56.97±3.37de	76.55±3.19cde	80.96±1.52ghi
	LYX73	73.9±0.76a	75.21±2.95cdef	84.88±1.23def

Note: Different lower case letters in the same column mean that there is a significant difference in the nematicidal activity of different combinations in the same treatment time (means were compared by Duncan's multiple range test at $p < 0.05$)

Table S5. Nematicidal activity of Fungal fermentation filtrate compounded with Green substances.

Compounding Agent		24h corrected mortality rate (%)	48h corrected mortality rate (%)	72h corrected mortality rate (%)
1.5g/L Arecoline	LYZ21	18.78±3.69i	54.67±11.50hi	88.65±2.70de
	CSUFT-F23	75.39±2.02cd	81.53±1.32cde	89.18±1.30de
	CSUFT-F32	45.63±4.44f	62.26±3.48g	70.75±3.40hi
	CSZ33	81.02±1.06bc	88.37±4.10ab	95.72±3.95ab
	CSUFT-F7	21.35±1.26hi	54.86±3.82hi	74.54±7.13gh
	CSZ71	88.71±3.08a	90.49±1.11ab	94.93±4.44abc
1.5g/L Sodium silicate	LYZ21	80.04±2.64bc	80.78±4.38cde	89.62±4.49de
	CSUFT-F23	90.51±2.84a	92.30±2.40a	95.88±3.59a
	CSUFT-F32	60.42±4.04e	68.68±5.71f	78.47±4.41fg
	CSZ33	61.60±1.72e	85.51±0.89bc	90.59±1.62bcde
	CSUFT-F7	44.91±14.51f	60.34±4.43gh	68.45±4.74i
	CSZ71	75.43±2.24cd	79.02±5.79de	92.70±0.99abcd
1.5g/L Ginkgo biloba extract	LYZ21	26.50±2.58gh	59.14±2.15gh	68.79±2.75i
	CSUFT-F23	71.73±4.28d	76.14±1.73e	86.51±1.08e
	CSUFT-F32	28.14±1.69g	51.98±3.10i	80.70±3.36f
	CSZ33	62.04±3.04e	80.89±1.69cde	90.25±1.13cde
	CSUFT-F7	29.75±1.81g	57.90±3.86ghi	78.11±1.49fg
	CSZ71	82.11±2.59b	85.02±0.60bcd	88.78±1.89de

Note: Different lower case letters in the same column indicate significant differences in the nematicidal activities among the various compound combinations within the same treatment time (means were compared by Duncan's multiple range test at $p < 0.05$)

Table S6. Compound combination kill rate

Compounding Agent		24h corrected mortality rate (%)	48h corrected mortality rate (%)	72h corrected mortality rate (%)
Arecoline	CSX134	69.89±2.63	85.92±4.43	94.38±0.61
	CSZ33	81.02±1.06	88.37±4.10	95.72±3.95
	CSZ71	88.71±3.08	90.49±1.11	94.93±4.44
Sodium silicate	CSUFT-F23	90.51±2.84	92.30±2.40	95.88±3.59
	CSZ71	75.43±2.24	79.02±5.79	92.70±0.99

Note: Different lower case letters in the same column indicate significant differences in the nematicidal activities among the various compound combinations within the same treatment time (means were compared by Duncan's multiple range test at $p < 0.05$)

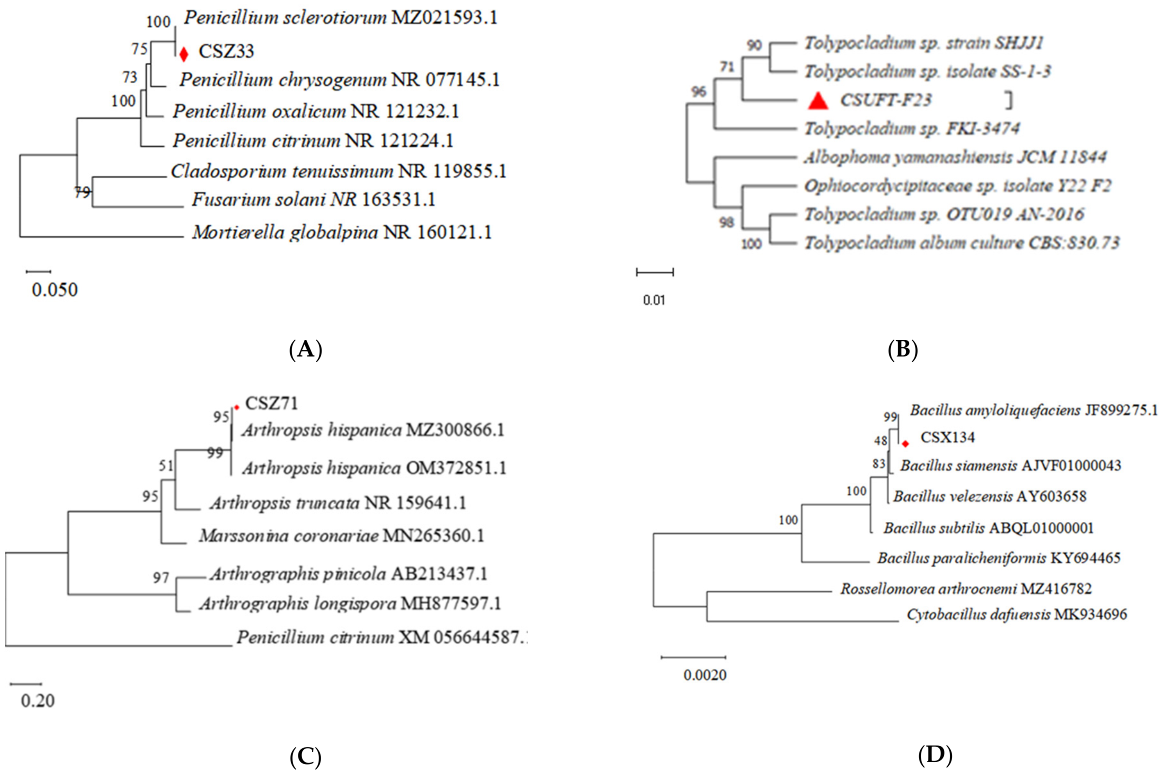

Figure S2. ITS-rRNA phylogenetic tree. Note: (**A**): CSZ33; (**B**): CSUFT-F23; (**C**): CSZ71; (**D**): CSX134.

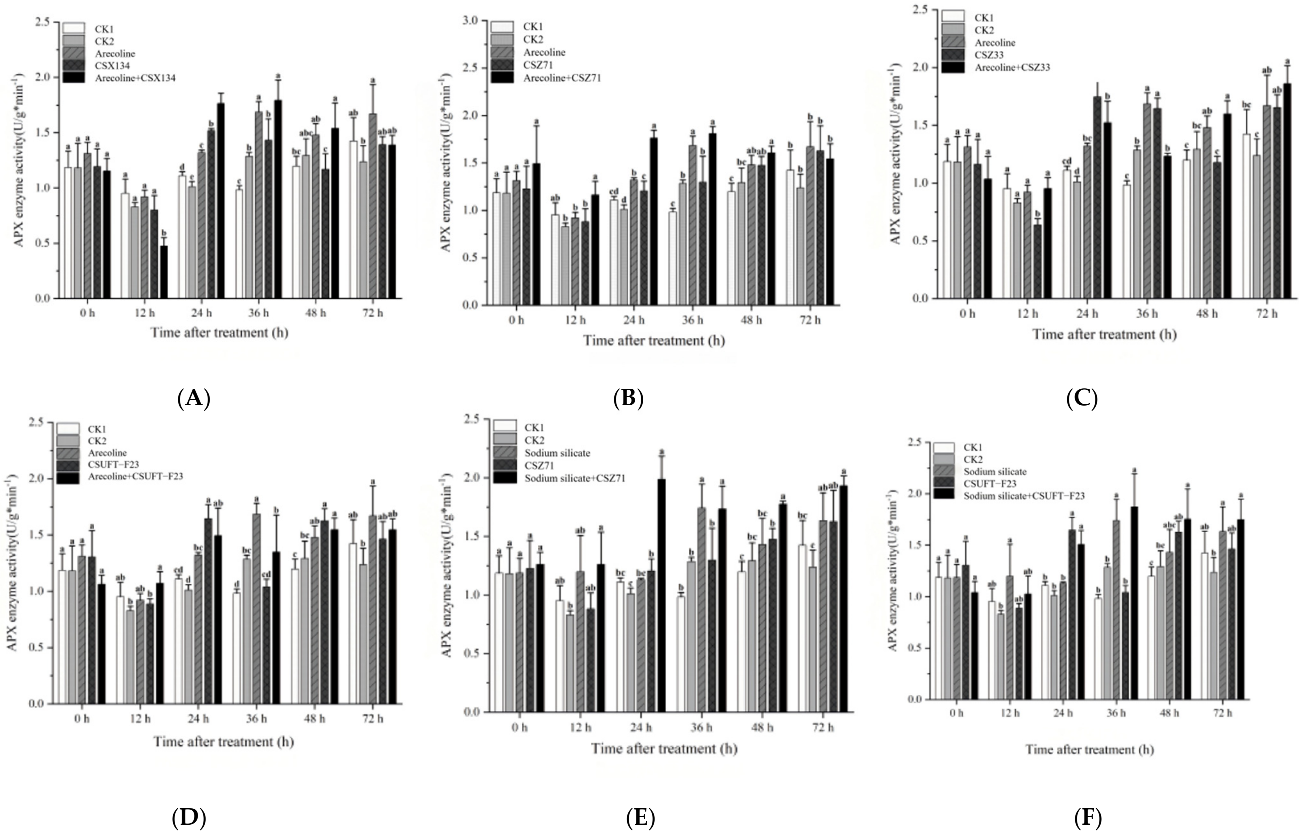

Figure S3. The changes in APX activity within *Pinus massoniana* seedlings under different treatments over 72 hours. Note: (**A**): arecoline plus CSX134 composite treatment; (**B**): arecoline plus CSZ71 composite treatment; (**C**): arecoline plus CSZ33 composite treatment; **(D**): arecoline plus CSUFT-F23 composite treatment; (**E**): sodium silicate plus CSZ71 composite treatment; (**F**): sodium silicate plus CSUFT-F23 composite treatment; CK: treated with sterile water; CK1: treated with *B. xylophilus* control; lowercase letters indicate significant differences in the activity of APX in *P. massoniana* under different treatments at the same time point (means were compared with Duncan's multiple range test at $p < 0.05$).

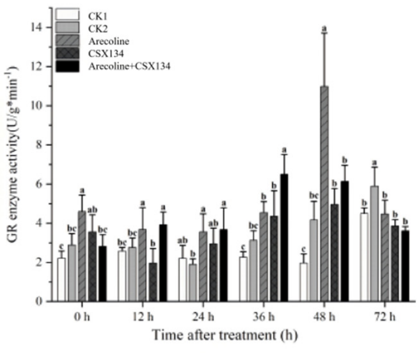

(**A**)

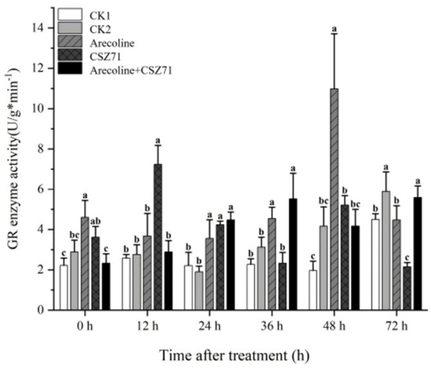

(**B**)

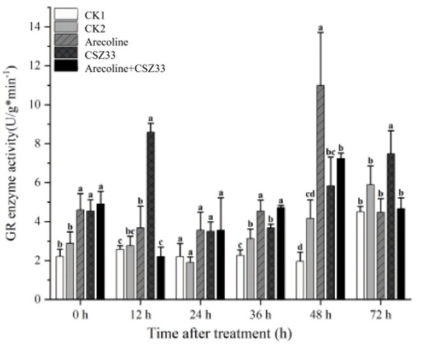

(**C**)

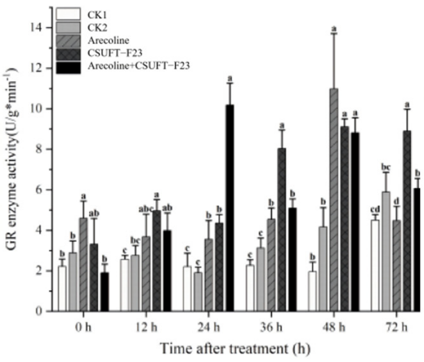

(**D**)

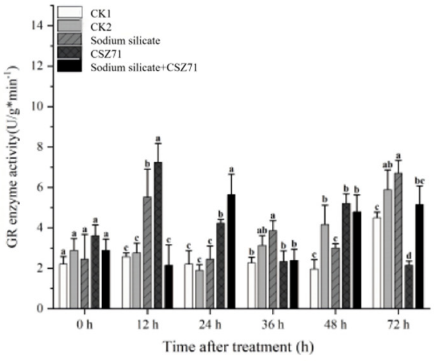

(**E**)

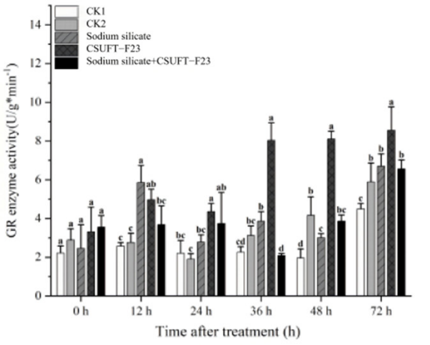

(**F**)

Figure S4. The changes in GR activity within *P. massoniana* seedlings under different treatments over 72 hours. Note: (**A**): arecoline plus CSX134 composite treatment; (**B**): arecoline plus CSZ71 composite treatment; (**C**): arecoline plus CSZ33 composite treatment; (**D**): arecoline plus CSUFT-F23 composite treatment; (**E**): sodium silicate plus CSZ71 composite treatment; (**F**): sodium silicate plus CSUFT-F23 composite treatment; CK: treated with sterile water; CK1:treated with *B. xylophilus* control; lowercase letters indicate significant differences in the activity of GR in *P. massoniana* under different treatments at the same time point (means were compared with Duncan's multiple range test at $p < 0.05$).

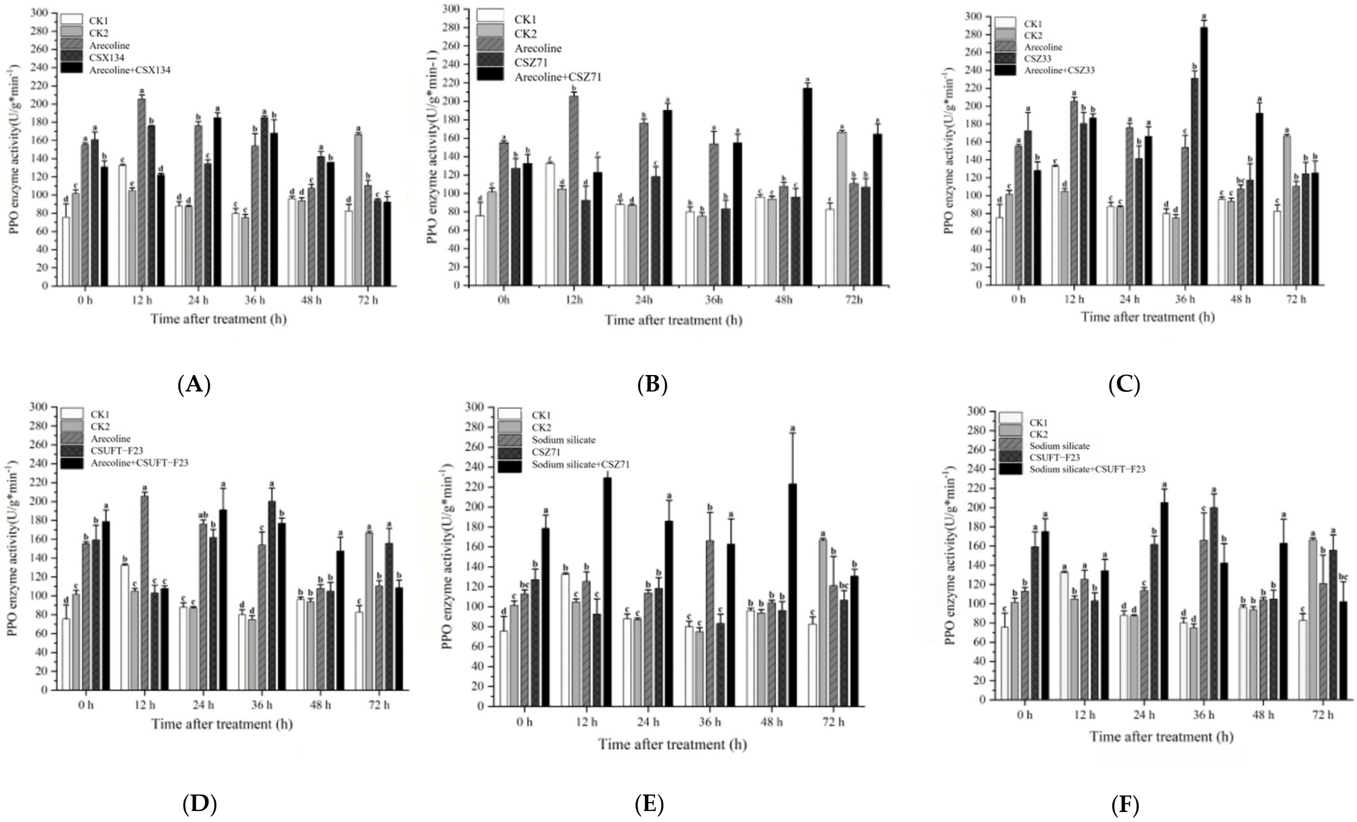

Figure S5. The changes in PPO activity within *P. massoniana* seedlings under different treatments over 72 hours. Note: (**A**): arecoline plus CSX134 composite treatment; (**B**): arecoline plus CSZ71 composite treatment; (**C**): arecoline plus CSZ33 composite treatment; (**D**): arecoline plus CSUFT-F23 composite treatment; (**E**): sodium silicate plus CSZ71 composite treatment; (**F**): sodium silicate plus CSUFT-F23 composite treatment; CK: treated with sterile water; CK1:treated with *B. xylophilus* control; lowercase letters indicate significant differences in the activity of PPO in *P. massoniana* under different treatments at the same time point (means were compared with Duncan's multiple range test at $p < 0.05$).

Table S7. Effect of different treatments on the control of pine wilt disease in *P. massoniana*

Treatments	Disease Index(%)	Biocontrol Efficacy(%)
CK（Pine wood nematode inoculation）	86.25±3.31a	-
Arecoline	40.00±5.73e	53.62±6.64b
Sodium silicate	42.08±3.15de	51.21±3.64bc
CSX134	47.50±3.75cde	44.93±4.35bcd
CSZ71	55.83±6.29b	35.27±7.29d
CSZ33	52.92±5.91bcd	38.65±6.85cd
CSUFT-f23	49.58±4.39cde	42.51±5.09bcd
Arecoline+CSX134	46.25±9.76cde	46.37±11.32bcd
Arecoline+CSZ71	45.00±9.92bcd	47.82±11.50bcd
Arecoline+CSZ33	20.00±5.00f	76.81±5.80a
Arecoline+CSZ23	43.33±5.91cde	49.76±6.85bcd
Sodium silicate+CSZ71	54.17±15.83bc	37.20±18.35cd
Sodium silicate+CSUFT-f23	23.33±6.88f	72.95±7.98a

Note: Different lower-case letters in the same column mean that there is a significant difference in the effectiveness of pine nematode control between different treatments within the same treatment time (means were compared by Duncan's multiple range test at $p < 0.05$).
