# Supplementary material for: Enhanced Efficacy of Rhizosphere Microorganisms and Green Compounds: A Dual-Action Strategy Against Bursaphelenchus xylophilus in Pinus massoniana
Source: Microorganisms. 2026 May 26;14(6):1202. doi: 10.3390/microorganisms14061202 (PMC13303170; doi:10.3390/microorganisms14061202)
Supplement: Supplementary file 1 [file microorganisms-14-01202-s001.zip › Supplementary materials file S2. Methods in detail.pdf]

Enhanced Efficacy of Rhizosphere Microorganisms and Green Compounds: A Dual-action Strategy Against *Bursaphelenchus xylophilus* in *Pinus massoniana*

Jiacheng Zhu[1], Yi Dang[2], Xiaoming Ren [3],Long Xu[3] ,Yilong Zhou[4] ,Guoying Zhou [1,2,3,4]*, and Junang Liu [1,2,3,4]*

1 College of Forestry, Central South University of Forestry and Technology, Changsha 410004, China
2 Hunan Provincial Key Laboratory for Control of Forest Diseases and Pestsand Pests in South China, Central South University of Forestry and Technology, Changsha 410004, China
3 Key Laboratory for Non-Wood Forest Cultivation and Conservation of Ministry ofEducation, Central South University of Forestry and Technology, Changsha 410004, China
4 Yuelushan Laboratory Non-wood Forests Variety Innovation Center, Central South University of Forestry and Technology, Changsha 410004, China
* Correspondence: zgyingqq@163.com (G.Z.); kjc9620@163.com (J.L.)

1. Methods

1.1 Culturing and Isolation of Bursaphelenchus xylophilus

After sterilising the laminar flow hood for 20 minutes, ventilate it for 5 minutes. Heat the solid PDA medium in a microwave oven until liquefied, then pour it into 90 mm culture plates. Once the medium has solidified, inoculate the PDA medium with *Botrytis cinerea*, seal with a lid, and incubate in a dark incubator at 25 °C until the mycelium completely covers the surface of the medium. Introduce the pine wood nematode into the petri dish colonised by *B. cinerea*. After 5 to 7 days, when the pine wood nematode has consumed all the mycelium, isolate and collect the nematodes. Pine wood nematodes were isolated using the Belman funnel method. The collected nematode suspension was centrifuged at 3,500 rpm for 5 minutes. Following centrifugation, the supernatant was removed, and the remaining pine wood nematodes were washed three times with sterile water before being prepared as a nematode suspension.

1.2 Culture medium formulation

Table S8 Related media formulations

Test medium	Liquid Media Formulation
Luria-Bertani（LB）	LB medium: 10 g tryptone, 5 g yeast extract, and 10 g NaCl were dissolved in 1000 mL of sterile water. For solid medium, 20 g of agar was added.
Potato Dextrose Agar（PDA）	PDA medium: 200 g of potato and 20 g of glucose were used per 1000 mL of sterile water. Agar (20 g) was added when preparing solid medium.

1.3 Green agent pot trial on Pinus massoniana inoculated with B. xylophilus

The treatment groups were arranged as follows:

Single-agent treatments: During the initial 72 h period, each treatment received 50 mL of either sodium silicate (1.5 g/L), arecoline (1.5 g/L), or fermentation filtrates of CSX134, CSZ71, CSZ33, or CSUFT-F23. Both control groups (CK and CK1) received 50 mL of sterile water. After three days, all treatment groups except CK were inoculated with 2 mL of nematode suspension (approximately 3000 nematodes/mL), while CK received 2 mL of sterile water.

Combined-agent treatments: Each combined treatment received 50 mL of a 1:1 mixture, consisting of 25 mL of the fermentation filtrate and 25 mL of the corresponding green agent. The combinations tested were arecoline + CSX134, arecoline + CSZ71, arecoline + CSZ33, arecoline + CSUFT-F23, sodium silicate + CSZ71, and sodium silicate + CSUFT-F23. Each treatment was applied to three seedlings.

To prevent the spread of pine wood nematodes, all materials used in the experiment were sterilized by autoclaving before disposal.

1.4 Assay of Defense Enzyme and MDA Activities in P. massoniana Seedlings

All assays were performed following the instructions provided with the respective commercial kits.

For APX activity, approximately 0.1 g of P. massoniana needles was homogenized with the reagents specified in the kit. Absorbance was measured at 290 nm, with readings taken at 30 s and again at 5 min 30 s.

For GR activity, 0.1 g of needle tissue was mixed with the assay reagents. Absorbance was recorded at 412 nm immediately after mixing (A1 at 30 s) and again after a 10 min incubation at room temperature (A2).

PPO activity was assessed using 0.1 g of needles. After mixing with the kit reagents, absorbance was measured at 420 nm immediately (A1) and after a 5 min incubation (A2).

MDA content was determined using 0.1 g of needle tissue. The sample was mixed with the reagents and incubated in a 90 – 95 ° C water bath for 30 min, then cooled on ice and centrifuged at 12,000 rpm for 10 min at 25 ° C. The supernatant was transferred to a 1 mL glass cuvette, and absorbance was measured at 532 nm and 600 nm.

Detailed procedures for each assay are available in the respective kit manuals.

1.5 Criteria for grading pine wood nematode diseases

Table S9 Criteria for grading *B. xylophilus* diseases

**Disease severity**	**Classification criteria**	**Disease scoring**
0	Needles are green, and branches appear normal.	0
I	Needles begin to yellow, affecting up to one quarter of the foliage.	1
II	Yellowing extends beyond one quarter but remains less than three quarters of the foliage.	2
III	More than three quarters of the needles are yellow; up to half show reddening and early wilting.	3
Ⅳ	Needles are completely red, with over half showing wilting; branches are moribund or dead.	4

1.6 qPCR Primers for mRNA

Table S10 Sequences of qPCR primers for mRNAs

Gene	Forward primer	Reverse primer
PmSOD	ATGGCCACTATCCGTTTCAG	TGCGAGAGAGTGACAACACC
PmCAT	ATTCTTGGGGTGACAGTTGC	TTTGTTGAACTGCGCAAGAC
P450 Ctochrome P450	GTCGGAAACCTCCACCAAC	TAGGGACTGAGCCCAAGC
PR-2(β-1,3-glucanase)	CGACAACATTCGCCCCTTCT	CTGCAGCGCGGTTTGAATAT
PR-3(class l chitinase)	ACCTACAGCGCCTTCATTGC	TGTGGTTTCATGCGACGTTT
Actin	CCTTGGCAATCCACATC	TCACCACTACGGCAGAAC

1.7 Statistical Analysis

Mortality in the *B. xylophilus* assay was measured and then corrected using the Chandravadana method. Data were handled in Microsoft Office Excel 2016. Statistical analysis was performed with SPSS Statistics 19.0. One-way ANOVA tested for overall differences among groups. When a significant difference was found, means were separated by Duncan’s multiple range test at $p < 0.05$.
